# COL1A1-induced LOXL2 promotes ovarian cancer metastasis via a feedback loop upon inhibiting EGFR lysosomal degradation

**DOI:** 10.1038/s12276-026-01675-6

**Published:** 2026-03-11

**Authors:** Zhangjin Shen, Lingkai Gu, Mengxia Zheng, Yuwan Liu, Shanliang Shang, Yunshan Zhu, Weiguo Lu

**Affiliations:** 1 Department of Obstetrics and Gynecology, Sir Run Run Shaw Hospital, Zhejiang University School of Medicine, Hangzhou, China; 2 Zhejiang Key Laboratory of Maternal and Infant Health, Women’s Hospital, Zhejiang University School of Medicine, Hangzhou, China; 3 Zhejiang Key Laboratory of Precise Protection and Promotion of Fertility, Hangzhou, China; 4 Department of Gynecologic Oncology, Women’s Hospital, Zhejiang University School of Medicine, Hangzhou, China

**Keywords:** Ovarian cancer, Cell invasion

## Abstract

Widespread peritoneal metastasis is a key reason for high mortality in ovarian cancer and understanding its mechanisms will offer new targets for the effective treatment of metastatic ovarian cancer. Our previous research revealed that collagen type I alpha 1 (COL1A1)-induced upregulation of lysyl oxidase-like 2 (LOXL2) was markedly overexpressed in ovarian cancer tissue samples and ascites. LOXL2 serves as an independent risk factor for predicting prognosis and its high expression is associated with high-risk clinical factors. LOXL2 promoted migration and invasion of ovarian cancer cells and metastasis of transplanted tumors in the xenograft mouse model. Here we determined that, mechanistically, COL1A1 activated the EGFR–MEK–ERK signaling pathway, which subsequently facilitated the nuclear translocation of SP1. SP1 binds to the promoter region of LOXL2, augmenting its transcription. Meanwhile, LOXL2 interacts with EGFR, protecting it from lysosomal degradation and enhancing protein stability. Finally, EGFR activates the MEK–ERK signaling pathway, promoting the translocation of SP1 and forming positive feedback. Our findings confirmed the molecular mechanism by which COL1A1-induced upregulation of LOXL2 promotes ovarian cancer metastasis through a positive feedback loop involving EGFR–MEK–ERK–SP1, offering novel scientific insights and potential therapeutic targets for inhibiting ovarian cancer metastasis.

## LOXL2 drives ovarian cancer metastasis through a feedback loop

Epithelial ovarian cancer is a deadly form of cancer often diagnosed at an advanced stage due to vague early symptoms. Here researchers found that a protein called LOXL2 (lysyl oxidase-like 2) plays a key role in the spread of ovarian cancer. They discovered that another protein, COL1A1 (collagen type I alpha 1), increases LOXL2 levels, which helps cancer cells move and invade other tissues. Using human ovarian cancer cells and mice to study this process they found that when COL1A1 is present, it activates a pathway inside the cells that increases LOXL2 production. This makes the cancer more aggressive. They also showed that blocking LOXL2 can reduce the spread of cancer in mice. The study suggests that targeting LOXL2 could be a new way to treat ovarian cancer and prevent it from spreading.

This summary was initially drafted using artificial intelligence, then revised and fact-checked by the author.

## Introduction

Epithelial ovarian cancer is the most lethal gynecological malignant tumor worldwide^1^. Extensive peritoneal and distant organ metastases in advanced ovarian cancer contributes to its high mortality rate^2^. As the ovaries are located deep in the pelvic cavity, ovarian cancer is highly insidious. Most patients with ovarian cancer are diagnosed at an advanced stage and have a poor prognosis owing to atypical symptoms in the early stages^3^. The preferred treatment for patients with advanced ovarian cancer is debulking surgery combined with carboplatin and paclitaxel chemotherapy^4^; however, over 75% of patients experience disease recurrence, chemotherapy resistance, and other challenges within 2–3 years of the initial treatment, leading to an unfavorable prognosis^5^. Consequently, understanding the molecular mechanisms underlying ovarian cancer metastasis is crucial for improving treatments.

In our previous study, we discovered that collagen type I alpha 1(COL1A1) secreted by fibroblasts enhanced the migration and invasion of ovarian cancer cells^6^. To reveal the molecular mechanisms promoting ovarian cancer metastasis, we conducted a proteomics analysis of ovarian cancer cells treated with and without COL1A1 and observed that COL1A1 reshaped the microenvironment of ovarian cancer. We focused on lysyl oxidase-like 2 (LOXL2), a secreted protein significantly upregulated in ovarian cancer cells incubated with COL1A1. LOXL2, a copper-dependent amine oxidases belonging to the lysyl oxidase family, catalyzes the crosslinking of elastin and collagen in the extracellular matrix^7^. The distinctive functions of LOXL2 in numerous conditions, such as liver fibrosis, have been clarified^8^. In addition to its role in the extracellular matrix, LOXL2 plays a critical role within cells. Recently, an increasing number of studies have shown that abnormal LOXL2 expression in multiple cancers is associated with chemoradiotherapy resistance^9,10^, metastasis^11^ and poor prognosis^12^. Aberrant LOXL2 expression in cancer is regulated by multiple pathways, such as microRNA regulation^8^, hypoxic tumor microenvironment^13^, transcriptional regulation^14^, mRNA stability regulation^15^ and post-translational modification^16^. However, the role and mechanism of LOXL2 in ovarian cancer metastasis remain unclear.

In the present study, we found that COL1A1 significantly promoted high LOXL2 expression in ovarian cancer cells. In addition, LOXL2 expression was dramatically upregulated in ovarian cancer tissues and patients with higher LOXL2 expression had poorer prognoses. Functionally, LOXL2 promoted the migration, invasion and metastasis of ovarian cancer cells in a xenograft mouse model. Mechanistically, COL1A1 enhanced LOXL2 transcription by facilitating the nuclear translocation of SP1 through activation of the EGFR–MEK–ERK signaling pathway. LOXL2 binds to EGFR, protecting it from lysosomal degradation, thereby activating the MEK–ERK pathway in a positive feedback loop. To the best of our knowledge, our findings are the first to elucidate the role of LOXL2 in promoting ovarian cancer metastasis and its underlying mechanisms, potentially offering a novel therapeutic approach for the treatment of ovarian cancer metastasis.

## Materials and methods

### Cell culture

The human ovarian cancer cell lines CAOV3 and SKOV3 were obtained from the Women’s Reproductive Health Laboratory of Zhejiang Province. The cells were authenticated using DNA short tandem repeat (STR) profiling. SKOV3 cells were cultured in McCoy’s 5A medium with 10% fetal bovine serum (Everyday Green), penicillin (100 units/ml) and streptomycin (100 μg/ml). CAOV3 cells were cultured in Dulbecco’s modified Eagle medium with 20% fetal bovine serum (Thermo Fisher Scientific), penicillin (100 units/ml) and streptomycin (100 μg/ml). The cells were incubated at 37 °C in a 5% CO_2_ incubator in a humidified environment.

### Clinical specimens

This study was approved by the Ethics Committee of Women’s Hospital, School of Medicine, Zhejiang University (no. IRB-20210147-R). All tissue samples were collected at the Women’s Hospital, and informed consent was obtained from each patient before surgery. None of the patients had undergone radiotherapy or chemotherapy before surgery. The patient information is presented in Supplementary Table 1. All pathological diagnoses were reviewed by an expert pathologist.

### Animal studies

Four-to-six-week-old female BALB/c nude mice were used in the ovarian cancer xenograft mouse model. Luciferase-labeled cells (1.5 × 10^6^ cells in 100 μl PBS) were injected into the abdominal cavity of each mouse. Tumor development and metastasis were monitored using an in vivo imaging system (IVIS) (Lumina LT system, PerkinElmer) after peritoneal injection of 150 mg/kg D-luciferin. COL1A1 (2 μg in 200 μl PBS) was administered via intraperitoneal injection every other day and U0126 (15 mg/kg) was administered via intraperitoneal injection twice per week. After 4 weeks, the mice were killed and the numbers of metastatic tumors were counted to accurately assess peritoneal metastasis. Dissected tumor tissues were fixed in 4% paraformaldehyde and embedded in paraffin for immunohistochemical (IHC) staining. The investigators in all animal experiments were blinded to group allocation during animal treatment, data collection and outcome assessment. The animals were randomly assigned to their respective experimental groups, and each group was assigned a unique code. The key to the group codes was not revealed to the experimenters until all data collection and analysis were complete. All animal experiments were approved by the Animal Ethics and Welfare Committee of the Zhejiang Chinese Medical University (no. IACUC-20210607-11).

### Nuclear and cytoplasmic separation assay

The nuclear and cytoplasmic fractions of cells were separated using a Nuclear and Cytoplasmic Protein Extraction kit (Beyotime) according to the manufacturer’s instructions.

### Lentivirus, plasmid and siRNA transfection

LOXL2 and SP1 overexpression plasmids were constructed by overlapping PCR using primers designed with the Takara Primer design tool and transfected into cells using X-treme GENE HP DNA Transfection Reagent (Roche). LOXL2, SP1 and control small interfering (si)RNAs were synthesized by GenePharma. Transient transfection was performed using DharmaFECT Transfection Reagents (Thermo Fisher) in accordance with the standard protocol. LOXL2 overexpression, LOXL2 short hairpin (sh)RNA and negative control (NC) lentiviral vectors were synthesized by GeneChem and transduced into cells according to the manufacturer’s instructions. The target sequences of shRNAs and siRNAs are listed in Supplementary Table 2.

### RNA extraction and RT–qPCR analysis

Total RNA was extracted using TRIzol Reagent (Invitrogen). RNA was reverse transcribed using the PrimeScript RT Reagent kit with a gDNA eraser (TaKaRa). PCR was conducted using TB Green Premix Ex Taq (TaKaRa) and a 7900HT Fast Real-Time PCR system (Life Technologies). The primer sequences are listed in Supplementary Table 3. The relative mRNA expressions were calculated by the 2^−ΔΔ*Ct*^ method and normalized to GAPDH.

### Western blot analysis and immunohistochemistry

For western blot analysis, cells were lysed with RIPA and protein samples were separated by 10% SurePAGE gels (GenScript) and transferred to 0.22-μm PVDF membranes (Bio-Rad) using the eBlot L1 protein transfer system (GenScript). The membranes were blocked with 5% milk at room temperature and then imaged using an ImageQuant LAS 4000 Mini (Cytiva).

For IHC analysis, formalin-fixed and paraffin-embedded samples were first deparaffinized and rehydrated, and then washed with PBS. For antigen retrieval, slides were heated in a sodium citrate buffer at 95–100 °C for 8 min, followed by an 8-min period without heating. They were then reheated to 95–100 °C for 7 min and cooled to room temperature naturally. Subsequently, the slides were treated with 3% hydrogen peroxide for 25 min at room temperature, blocked using 5% bovine serum albumin (BSA) for 30 min and incubated with primary antibodies (listed in Supplementary Table 4) overnight at 4 °C. After that, they were incubated with a secondary antibody for 1 h at room temperature, stained with diaminobenzidine (DAB), counterstained with hematoxylin, dehydrated, mounted and finally imaged using K-Viewer software (version 1.7.0.27) or CaseViewer software (version 2.4.0.119028). The positive cells were scored as follows: 1, 0–25%; 2, 26–50%; 3, 51–75%; and 4, 76–100%. The staining intensity was scored as follows: 1, negative; 2, weak; 3, moderate; and 4, strong. The score for each microscopic field was obtained by multiplying the two scores and the sum was calculated by adding the scores of the three microscopic fields. The antibodies used in this study are listed in Supplementary Table 4.

### Immunofluorescence assay

Cells cultured on coverslips were washed with PBS, fixed with 4% formaldehyde for 20 min, permeabilized with 0.5% Triton X-100 for 20 min and blocked with 3% BSA for 1 h at room temperature. The cells were then treated with primary antibodies (listed in Supplementary Table 4) overnight at 4 °C and subsequently incubated with secondary antibodies conjugated with Alexa Fluor-488 or -555 for 1 h. The coverslips were washed with PBS, stained with DAPI (Abcam) and observed under a laser confocal microscope (Leica).

Paraffin sections were dewaxed to water using an eco-friendly dewaxing agent, absolute ethanol and distilled water. Antigen retrieval was performed with citrate buffer. After circling the tissue with a hydrophobic pen, the sections were incubated in 3% hydrogen peroxide solution in the dark for endogenous peroxidase blocking, followed by serum blocking. Subsequently, α-SMA antibody (Supplementary Table 4) was applied and incubated overnight in a humidified chamber. The corresponding HRP-conjugated secondary antibody was then added and incubated at room temperature, and TSA dye was added. The sections were then subjected to heat treatment in citrate buffer. Afterward, LOXL2 antibody (Supplementary Table 4) and its corresponding secondary antibody were applied. Finally, DAPI was used for nuclear counterstaining. Images were acquired using K-Viewer software (version 1.7.0.27) or CaseViewer software (version 2.4.0.119028).

### Transwell assay and wound healing assay

Twenty-four-well transwells (8-μm pore size, Corning) were used for evaluating cell invasion and migration ability. SKOV3 cells (1 × 10^5^/200 μl) and CAOV3 cells (2 × 10^5^/200 μl) were cultured in serum-free medium in the upper chambers, with or without Matrigel (Corning), and medium containing 10% FBS was added into the bottom chambers. Noninvasive or nonmigratory cells were removed after 6 or 24 h, and penetrated cells were fixed, stained and counted.

SKOV3 cells (5 × 10^4^) and CAOV3 (1 × 10^5^) cells were suspended in 80 μl of complete medium and seeded in Ibidi culture inserts (Ibidi). When the cells were fully confluent, the Ibidi culture inserts were removed to create a gap and serum-free medium was subsequently added. Images were captured using a microscope at 0 and 12 or 24 h. The distance between the gaps was measured, data were collected from three independent experiments and the migration rate of the cells was expressed as relative gap closure.

### ChIP assay

Chromatin immunoprecipitation (ChIP) assays were performed using a Magna ChIP A/G Chromatin Immunoprecipitation kit (17-10085; Merck Millpore) according to the manufacturer’s instructions. Briefly, cells were crosslinked with 1% formaldehyde for 15 min at room temperature and the chromatin was sonicated to generate DNA fragments. The sonicated lysates were immunoprecipitated with antibody (anti-SP1 or anti-IgG) at 4 °C overnight with magnetic protein A/G beads. Chromatin DNA was eluted from the protein–DNA complexes and purified using DNA purification columns and collection tubes. Purified DNA was analyzed by RT–qPCR. The ChIP primer sequences are listed in Supplementary Table 3. The PCR products were mixed with 5× prestained loading buffer, resolved electrophoretically on a 2% agarose gel and visualized under UV light

### Dual-luciferase reporter assay

The sequence of the LOX2 promoter region was cloned into a plasmid synthesized by GeneChem containing firefly luciferase. For the dual-luciferase reporter assay, ovarian cancer cells were transfected with the Renilla luciferase control vector, lysed with 1× cell lysis buffer at room temperature for 5 min and luciferase activity was determined using the Dual-Luciferase Reporter Assay kit (Yeasen) according to the manufacturer’s protocol. Relative luciferase activity was determined by calculating the ratio of firefly to Renilla fluorescence.

### Phalloidin staining

Cells receiving different treatments and cultured on coverslips were washed with PBS, fixed with 4% formaldehyde for 10 min, permeabilized with 0.5% Triton X-100 for 5 min and stained with fluorescein isothiocyanate (FITC)-phalloidin at room temperature for 30 min. Finally, the cells were stained with DAPI for 30 s and observed by laser confocal microscopy (Leica).

### Statistical analysis

Statistical analyses were conducted using SPSS 27.0 (IBM) and GraphPad Prism 9.0 software (GraphPad Software). All experiments were performed in triplicate. Measured data are represented as the mean ± s.d. A one-way analysis of variance (ANOVA) or two-tailed Student’s *t*-test was applied to compare quantitative data, while the nonparametric χ2 test was used to analyze qualitative data. *P* values for each analysis are marked on the figures, and the level of statistical significance was defined as *P* < 0.05 (*P < 0.05, **P < 0.01, ***P < 0.001 and ****P < 0.0001).

## Results

### LOXL2 acts as an oncogene and a downstream target of COL1A1

**Fig. 1:**
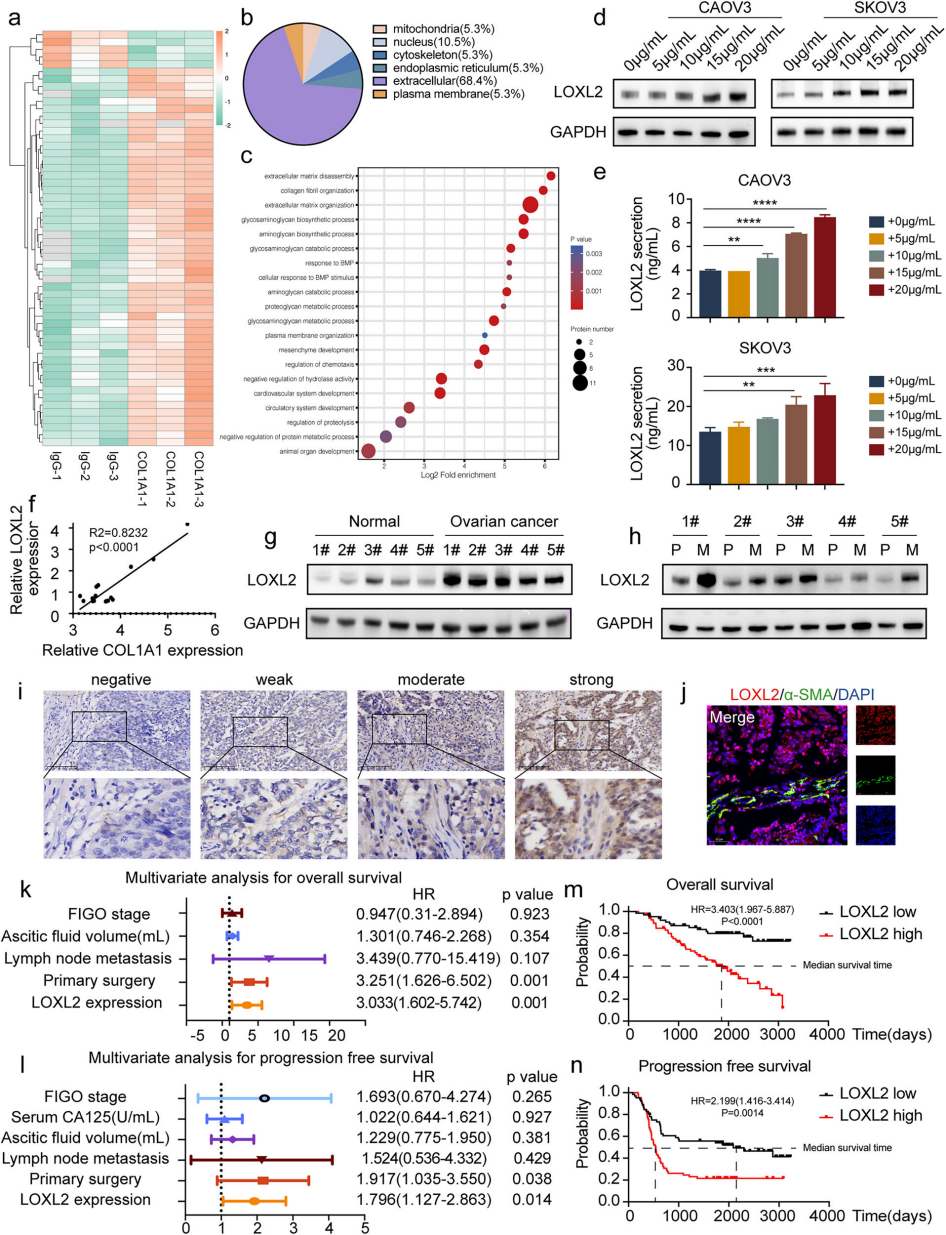
LOXL2 acts as an oncogene and a downstream target of COL1A1. **a** Differentially expressed proteins were compared between CAOV3 cells treated with IgG and COL1A1 by using LC–MS/MS label-free quantitative proteomics. **b** The subcellular localization of proteins differentially expressed following COL1A1 and IgG treatments. **c** GO analysis of proteins differentially expressed following COL1A1 and IgG treatments. **d**,**e** Immunoblot analysis (**d**) and ELISA (**e**) of LOXL2 in CAOV3 and SKOV3 cells with different concentrations of COL1A1 treatment. **f** The protein levels of COL1A1 and LOXL2 in 17 ovarian cancer ascites were detected by ELISA. **g** Immunoblot analysis of LOXL2 expression in ovarian cancer tissues and normal ovarian tissues. **h** Immunoblot analysis of LOXL2 expression in ovarian cancer tissues and matched metastatic ovarian cancer tissues. P: primary ovarian cancer tissues. M: metastatic ovarian cancer tissues. **i** Representative IHC staining images of LOXL2 in ovarian cancer tissues. Scale bar, 100 μm. **j** Representative images of immunofluorescence staining of α-SMA (green) and LOXL2 (red) in ovarian cancer tissues. Scale bar, 50 μm. **k**,**l** Multivariate Cox regression analysis of overall survival (**k**) and progression-free survival (**l**) in patients with ovarian cancer. HR, hazard ratio. **m**,**n** Kaplan–Meier analysis of overall survival (**m**) and progression-free survival (**n**) of 129 patients with ovarian cancer in our hospital. Data are representative of at least three independent experiments. *P < 0.05, **P < 0.01, ***P < 0.001 and ****P < 0.0001.

To understand the mechanism of COL1A1 in ovarian cancer, CAOV3 cells treated with and without COL1A1 were selected for protein expression profiling by liquid chromatography–tandem mass spectrometry LC–MS/MS label-free quantitative proteomics, and 51 upregulated proteins and 5 downregulated proteins (fold change >1.5 or <0.68, *P* < 0.05) were found in cells treated with COL1A1 (Fig. 1a). To more accurately understand the mechanism of COL1A1, we focused on exploring the upregulated proteins with more than a twofold change. Interestingly, most differentially expressed proteins were localized in the extracellular matrix (Fig. 1b), and Gene Ontology (GO) analysis suggested that COL1A1 could induce extracellular matrix remodeling (Fig. 1c). Therefore, we focused on secretory proteins, of which LOXL2 was identified as one of the most significantly upregulated following COL1A1 treatment (Supplementary Fig. 1). LOXL2 protein expression and secreted LOXL2 protein levels notably increased in a dose-dependent manner in ovarian cancer cells treated with COL1A1 (Fig. 1d,e). Therefore, in the subsequent experiments, we used 20 μg/ml of COL1A1.

To understand the clinical significance of LOXL2, LOXL2 protein expression was validated in ovarian cancer ascites fluid by ELISA and the results showed a significant positive correlation between COL1A1 and LOXL2 protein expression (Fig. 1f). Furthermore, we detected LOXL2 expression in tissue samples. LOXL2 protein expression was higher in ovarian cancer tissues than in normal ovarian tissues (Fig. 1g), and similar results were found in ovarian cancer from Gene Expression Omnibus (GEO) datasets (Supplementary Fig. 2a,b). In addition, LOXL2 protein expression was further increased in metastatic ovarian cancer tissues compared to that in the primary ovarian cancer they were derived from (Fig. 1h). Moreover, we retrospectively collected the clinicopathological data of 129 patients with ovarian cancer and their formalin-fixed and paraffin-embedded tissue samples, and immunohistochemistry (Fig. 1i) and immunofluorescence double staining (Fig. 1j) for LOXL2 protein revealed its expression in both cells and the extracellular matrix. Furthermore, a significant association was found between LOXL2 expression and International Federation of Gynecology and Obstetrics (FIGO) stage, lymph node metastasis, ascitic fluid volume and CA125 levels, as presented in Table 1. Univariate and multivariate analyses showed that LOXL2 expression was an independent prognostic factor for overall survival (OS) and progression-free survival (PFS) (Fig. 1k,l and Supplementary Fig. 3). Kaplan–Meier survival analysis also showed shorter progression-free survival and overall survival in patients with high LOXL2 expression than in those with low LOXL2 expression (Fig. 1m,n). We additionally analyzed the data in The Cancer Genome Atlas (TCGA) database and found the same results (Supplementary Fig. 4a,b). Taken together, our data suggest that LOXL2 acts as a downstream target of COL1A1 and that patients with higher LOXL2 expression have a poor prognosis.

**Table 1:** Clinical characteristics of 129 patients with ovarian cancer depending on their LOXL2 protein level.

variable	N	PLAA expression		*P* value
		Low	High	
Age (years)				
≤50	46	24	22	0.665
>50	83	40	43	
FIGO stage				
Ⅰ/Ⅱ	30	23	7	0.001^**^
Ⅲ/Ⅳ	99	41	58	
Lymph node metastasis				
Negative	25	19	6	0.003^**^
Positive	104	45	59	
Ascitic fluid volume (ml)				
<500	83	49	34	0.004^**^
≥500	46	15	31	
Serum CA125 (U/ml)				
<500	70	41	29	0.027^*^
≥500	59	23	36	
Tumor diameter (cm)				
<8	63	30	33	0.658
≥8	66	34	32	
Primary surgery				
Optimal	114	58	56	0.428
Suboptimal	15	6	9	

A χ^2^ Test was used to calculate the association between categorical variables. *P < 0.05 and **P < 0.01.

### LOXL2 promotes ovarian cancer migration and invasion in vitro and in vivo

**Fig. 2:**
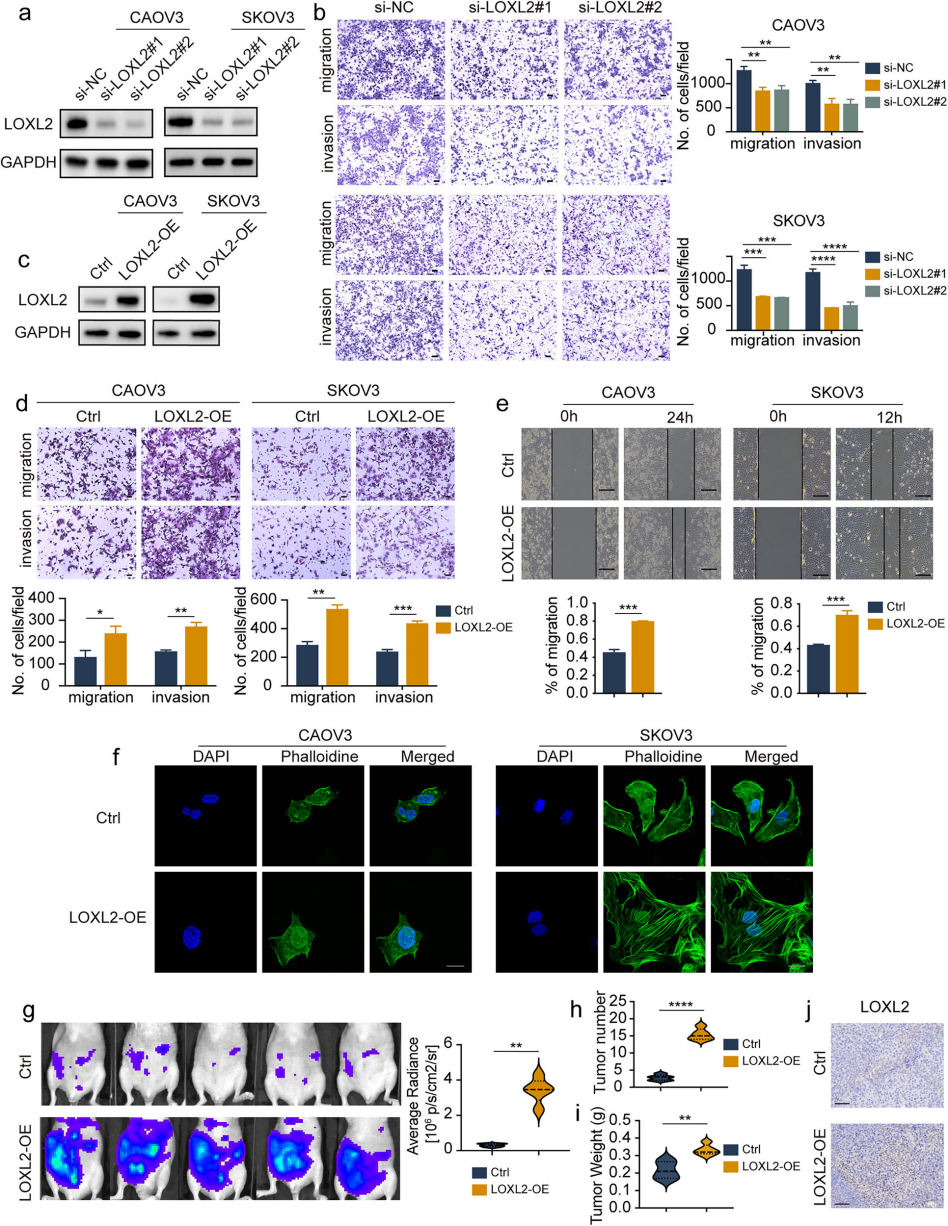
LOXL2 promotes ovarian cancer migration and invasion in vitro and in vivo. **a** CAOV3 and SKOV3 cells were transfected with two LOXL2 siRNAs or NC and LOXL2 expression was determined by immunoblot analysis. **b** CAOV3 and SKOV3 cells were transfected with two LOXL2 siRNAs or NC. Cellular migration and invasion were detected by a transwell assay. Scale bar, 100 μm. **c** CAOV3 and SKOV3 cells were transfected with LOXL2-OE plasmid or empty plasmid. LOXL2 expression was determined by immunoblot analysis. **d** CAOV3 and SKOV3 cells were transfected with LOXL2-OE plasmid or empty plasmid. Cellular migration and invasion were detected by a transwell assay. Scale bar, 100 μm. **e** CAOV3 and SKOV3 cells were transfected with LOXL2-OE plasmid or empty plasmid. Cellular migration was detected by a wound healing assay. Scale bar, 50 μm. **f** The cellular structure of CAOV3 and SKOV3 cells transfected with LOXL2-OE plasmid or empty plasmid were detected by phalloidin staining. Scale bar, 50 μm. **g**–**i** CAOV3-LOXL2-luc or CAOV3-control-luc cells were transplanted into mice with representative images and luminescence quantification of peritoneum metastasis of transplanted tumors at the endpoints (**g**), quantitative analysis of the number of metastatic tumors in mice (**h**) and measurement of metastatic tumor weight in mice (**i**). **j** Representative IHC staining images of LOXL2 in xenograft mouse model. Data are representative of at least three independent experiments. *P < 0.05, **P < 0.01, ***P < 0.001 and ****P < 0.0001.

To explore the biological function of LOXL2 in ovarian cancer, two specific siRNAs of LOXL2 were transfected into CAOV3 and SKOV3 cells, LOXL2 mRNA and protein expression was notably downregulated in both cell lines (Fig. 2a and Supplementary Fig. 5a). To evaluate the impact of LOXL2 on the migration and invasion abilities of ovarian cancer cells in vitro, wound healing and transwell assays were performed. Our results demonstrated that LOXL2 downregulation significantly decreased the migration and invasion abilities of both cell lines (Fig. 2b and Supplementary Fig. 5b). Lamellipodia are important regulators of the motility of tumor cells and are composed of F-actin, therefore phalloidin staining was used to observe the structure and distribution of F-actin. Our results revealed that LOXL2 knockdown dramatically decreased lamellipodia formation (Supplementary Fig. 5c).

We also transfected the LOXL2 plasmid into CAOV3 and SKOV3 cells and found that LOXL2 mRNA and protein expression were successfully upregulated (Fig. 2c and Supplementary Fig. 5d). Wound healing and transwell assays revealed that the upregulation of LOXL2 significantly increased wound closure, ovarian cancer cell migration and invasion ability (Fig. 2d,e). In addition, phalloidin staining revealed that LOXL2 overexpression increased the number of lamellipodia (Fig. 2f). However, we found that increasing or decreasing LOXL2 did not significantly affect the migration or invasion of normal ovarian cells (Supplementary Fig. 6a–d).

We investigated the effect of LOXL2 on ovarian cancer metastasis in vivo utilizing a mouse xenograft model. LOXL2-overexpressing (OE) or empty luciferase-labeled plasmids were stably transfected into CAOV3 cells (CAOV3-LOXL2-luc cells and CAOV3-control-luc cells). CAOV3-LOXL2-luc or CAOV3-control-luc cells were injected into the abdominal cavities of nude mice. An IVIS was used weekly to monitor tumor progression. After 4 weeks, the mice were killed, the number of metastases was determined and the tumor tissues were collected. We demonstrated that LOXL2 overexpression significantly increased metastasis in the xenograft model and reduced mouse survival compared to the control group (Fig. 2g and Supplementary Fig. 6e,f). In addition, LOXL2 overexpression increased the number and weight of peritoneal metastases relative to the control group (Fig. 2h,i). IHC staining revealed the efficiency of LOXL2 overexpression in tumor tissues (Fig. 2j). Taken together, our results demonstrate that LOXL2 acts as an oncogene in ovarian cancer.

### COL1A1 promotes ovarian cancer metastasis through LOXL2

**Fig. 3:**
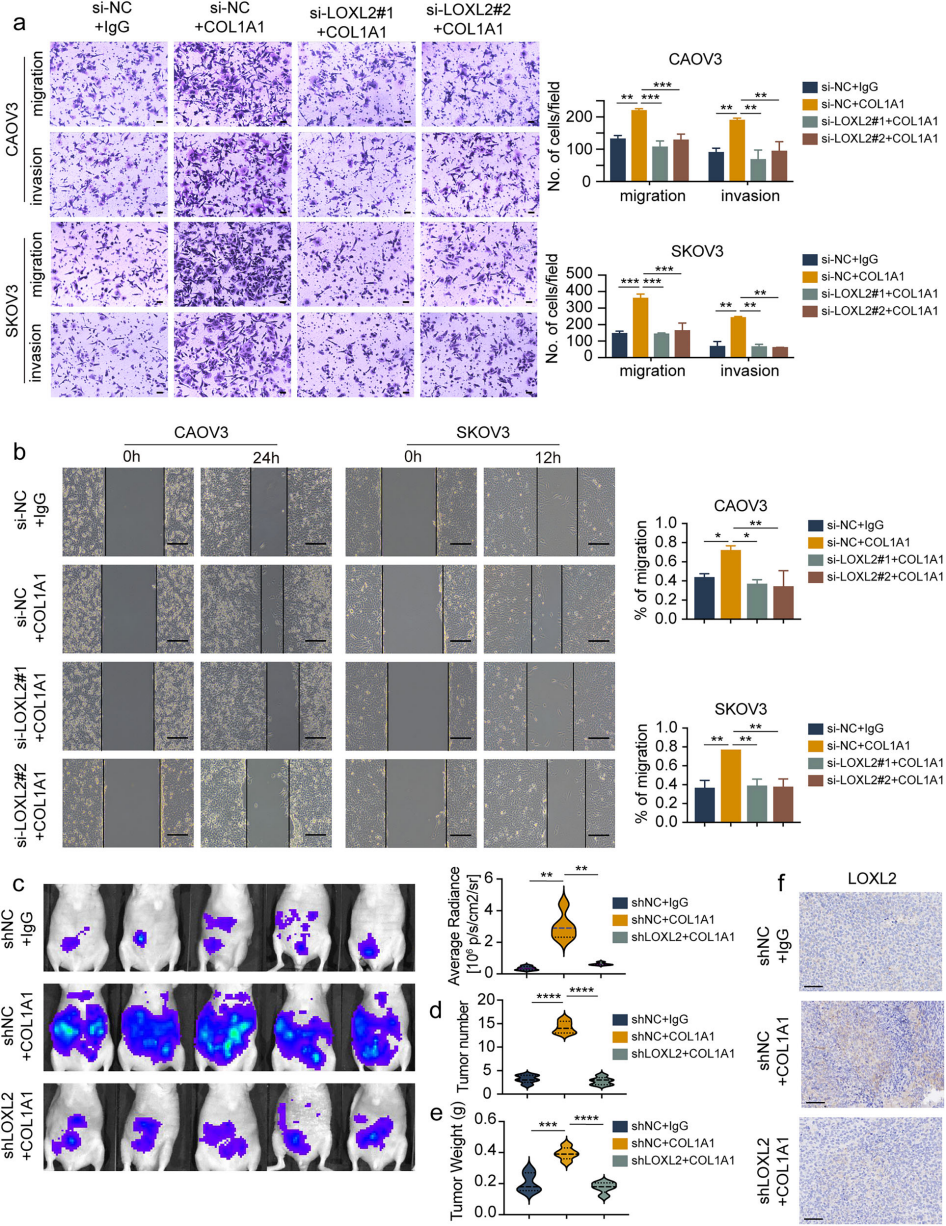
COL1A1 promotes ovarian cancer metastasis through LOXL2. **a** CAOV3 and SKOV3 cells were transfected with NC siRNA, NC siRNA plus COL1A1 treatment and LOXL2 siRNA plus COL1A1 treatment, respectively. Cellular migration and invasion were detected by a transwell assay. Scale bar, 100 μm. **b** CAOV3 and SKOV3 cells were transfected with NC siRNA, NC siRNA plus COL1A1 treatment and LOXL2 siRNA plus COL1A1 treatment, respectively. Cellular migration was detected by a wound healing assay. Scale bar, 50 μm. **c**–**e** Ovarian cancer cells with or without LOXL2 downregulation were transplanted into mice and mice were intraperitoneally treated with COL1A1 or IgG with representative images and luminescence quantification of peritoneum metastasis of transplanted tumors at the endpoints (**c**), quantitative analysis of the number of metastatic tumors in mice (**d**) and measurement of metastatic tumor weight in mice (**e**). **f** Representative IHC staining images of LOXL2 in xenograft mouse model. Data are representative of at least three independent experiments. *P < 0.05, **P < 0.01, ***P < 0.001 and ****P < 0.0001.

To investigate whether COL1A1 mediated ovarian cancer metastasis via LOXL2, we performed rescue assays. We found that LOXL2 downregulation significantly reversed the increased cell migration and invasion ability induced by COL1A1 (Fig. 3a). In addition, wound closure increased by COL1A1 was abrogated by LOXL2 siRNA in CAOV3 and SKOV3 cells (Fig. 3b).

Using the xenograft ovarian cancer mouse model, we injected CAOV3-shNC-luc (*n* = 10) or CAOV3-shLOXL2-luc (*n* = 5) cells into the abdominal cavities of nude mice and randomly divided the CAOV3-shNC-luc group into two subgroups (*n* = 5 per subgroup) 1 week after implantation. COL1A1 (2 μg per 200 μl) or IgG (2 μg per 200 μl) was intraperitoneally injected every other day from weeks 1 to 4, and the mice were euthanized after 4 weeks. To accurately assess the metastatic tumors, we measured the bioluminescent signal intensity in the peritoneal cavity using IVIS. We found that mice treated with COL1A1 showed wider metastases than those in the IgG group, which was abrogated by LOXL2 downregulation (Fig. 3c and Supplementary Fig. 7a). The increase in the number and weight of peritoneal metastases compared to that in controls was significantly abolished by LOXL2 downregulation (Fig. 3d,e). Moreover, IHC staining showed increased LOXL2 expression induced by COL1A1 in the xenograft mouse model tumor tissues (Fig. 3f). Our results together suggest that COL1A1 increases ovarian cancer metastasis in a LOXL2-dependent manner.

### COL1A1 facilitates LOXL2 transcription and expression via SP1

**Fig. 4:**
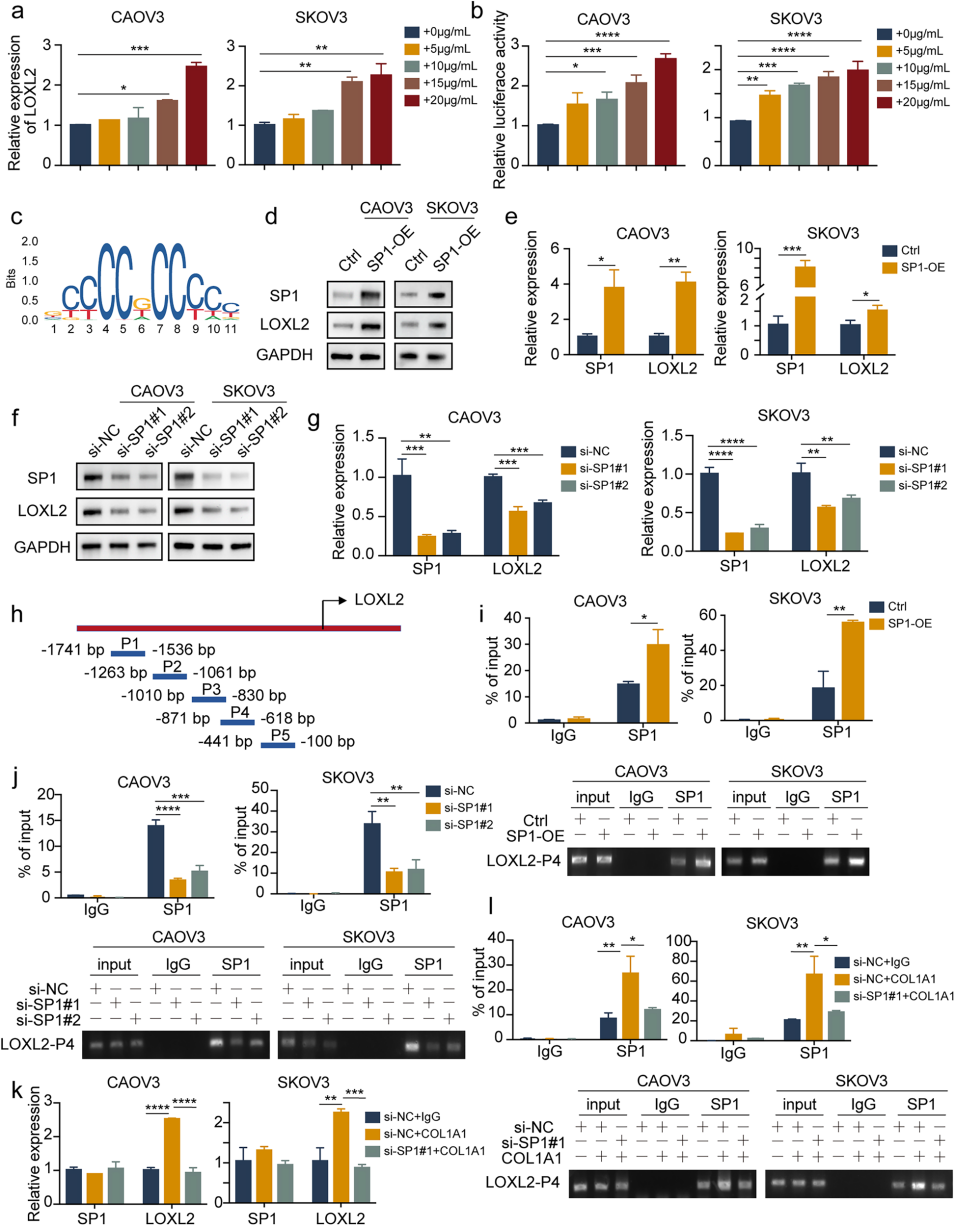
COL1A1 facilitates LOXL2 transcription and expression via SP1. **a** RT–qPCR analysis of LOXL2 in CAOV3 and SKOV3 cells with different concentrations of COL1A1 treatment. **b** Dual-luciferase reporter assays of LOXL2 transcription in CAOV3 and SKOV3 cells with different concentrations of COL1A1 treatment. **c** The putative binding site of SP1 in the LOXL2 promoter. **d**,**e** CAOV3 and SKOV3 cells were transfected with SP1 plasmid or NC, respectively. LOXL2 and SP1 expressions were determined by immunoblot analysis (**d**) and RT–qPCR (**e**). **f**,**g** CAOV3 and SKOV3 cells were transfected with SP1 siRNA or NC, respectively. LOXL2 and SP1 expressions were determined by immunoblot analysis (**f**) and RT–qPCR (**g**). **h** The primer design program for the LOXL2 promoter and its fragment in the ChIP assay. **i** CAOV3 and SKOV3 cells were transfected with SP1 plasmid or NC, respectively. Enrichment of SP1 or IgG at the LOXL2 promoter in ovarian cancer was assessed by a ChIP assay. **j** CAOV3 and SKOV3 cells were transfected with SP1 siRNA or NC, respectively. Enrichment of SP1 or IgG at the LOXL2 promoter in ovarian cancer was assessed by a ChIP assay. **k**,**l** CAOV3 and SKOV3 cells with or without SP1 knockdown were incubated with COL1A1, and LOXL2 and SP1 mRNA expression was determined by RT–qPCR (**k**) and enrichment of SP1 or IgG at the LOXL2 promoter was assessed by a ChIP assay (**l**). Data are representative of at least three independent experiments. *P < 0.05, **P < 0.01, ***P < 0.001 and ****P < 0.0001.

To further characterize the underlying mechanism by which COL1A1 increases LOXL2 protein expression, RT–qPCR was performed on CAOV3 and SKOV3 cells. Interestingly, our results indicated that with increasing concentrations of COL1A1, LOXL2 mRNA expression steadily increased, mirroring the trend observed at the protein level (Fig. 4a). Therefore, we hypothesized that COL1A1 promoted LOXL2 expression by increasing its transcription. The luciferase reporter assays consistently confirmed that COL1A1 promoted LOXL2 transcription in a dose-dependent manner (Fig. 4b).

We then sought to identify the transcription factors that influence LOXL2 transcription. We employed JASPAR (https://jaspar.elixir.no/) to discover whether the LOXL2 promoter contains putative binding sites for the classic transcriptional factor SP1, and the most common binding sites with high scores are shown in Fig. 4c. Consequently, we overexpressed SP1 by transfecting CAOV3 and SKOV3 cells with a SP1 plasmid and observed remarkably increased LOXL2 protein levels (Fig. 4d), mRNA levels (Fig. 4e) and transcriptional capacity (Supplementary Fig. 8a). By contrast, SP1 downregulation notably reduced LOXL2 protein levels (Fig. 4f), mRNA levels (Fig. 4g) and transcriptional capacity (Supplementary Fig. 8b).

To further confirm the precise binding site of SP1 in the LOXL2 gene promoter region, we designed five ChIP–qPCR primers for the LOXL2 promoter region, as shown in Fig. 4h. Our data showed that SP1 was able to bind to the promoters of LOXL2 in the −871 bp to −618 bp segment (Supplementary Fig. 8c). ChIP–qPCR detection further confirmed that SP1 overexpression in CAOV3 and SKOV3 cells significantly increased its binding enrichment to LOXL2 gene promoters (Fig. 4i). By contrast, SP1 knockdown decreased its binding to the promoters (Fig. 4j).

To investigate whether COL1A1 mediates LOXL2 expression in ovarian cancer cells through SP1, we added COL1A1 to ovarian cancer cells with or without SP1 downregulation. The increased expression of LOXL2 induced by COL1A1 was rescued by SP1 knockdown (Fig. 4k). Similarly, the binding enrichment ability between SP1 and LOXL2 accelerated by COL1A1 was reversed by SP1 downregulation (Fig. 4l). In conclusion, COL1A1 enhances LOXL2 expression by promoting its transcription via SP1.

### COL1A1 promotes SP1 translocation by activating the MEK–ERK pathway

**Fig. 5:**
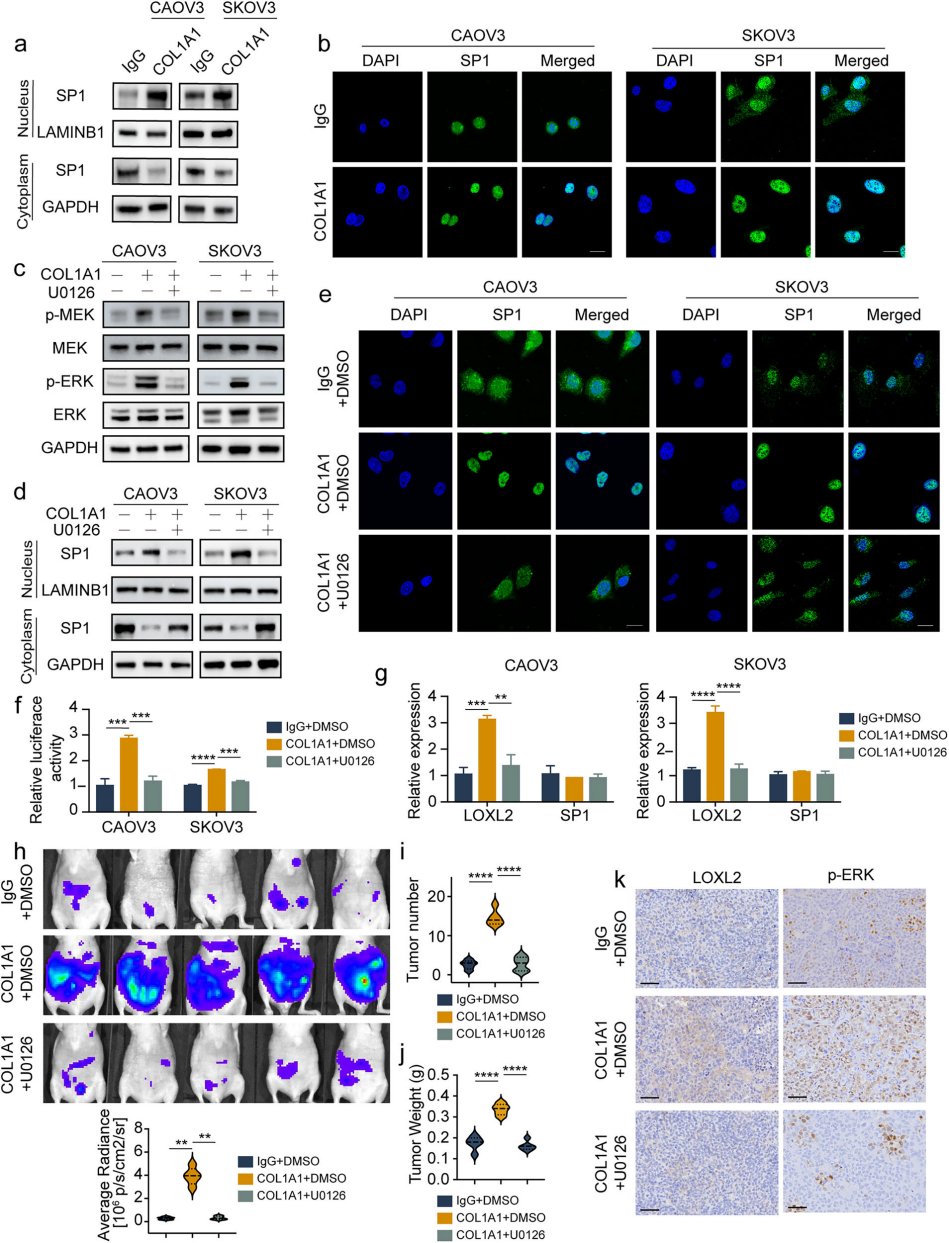
COL1A1 promotes SP1 translocation by activating the MEK–ERK pathway. **a,b** CAOV3 and SKOV3 cells were incubated with IgG or COL1A1, respectively, and SP1 translocation was determined by nuclear–cytoplasmic separation (**a**) and immunofluorescence (**b**). **c** CAOV3 and SKOV3 cells were treated with COL1A1, COL1A1 plus U0126 and NC, respectively. p-MEK, MEK, p-ERK and ERK expression was determined by immunoblot analysis. **d**,**e** SP1 translocation was determined by nuclear–cytoplasmic separation (**d**) and immunofluorescence (**e**) in ovarian cancer cells treated with COL1A1, COL1A1 combined with U0126 or NC. **f**,**g** CAOV3 and SKOV3 cells were treated with COL1A1, COL1A1 plus U0126 and NC, respectively. LOXL2 transcription were determined by dual-luciferase reporter assays (**f**) and LOXL2 and SP1 expression was determined by RT–qPCR (**g**). **h**–**j** Nude mice were treated with COL1A1, COL1A1 plus U0126 or NC, with representative images and luminescence quantification of peritoneum metastasis of transplanted tumors at the endpoints (**h**) quantitative analysis of the number of metastatic tumors in mice (**i**) and measurement of metastatic tumor weight in mice (**j**). **k** Representative IHC staining images of LOXL2 and p-ERK in xenograft mouse model. Data are representative of at least three independent experiments. *P < 0.05, **P < 0.01, ***P < 0.001 and ****P < 0.0001.

We measured SP1 protein levels in ovarian cancer cells incubated with or without COL1A1; however, no significant changes in SP1 levels were detected (Supplementary Fig. 9a). Considering that COL1A1 did not affect SP1 mRNA or protein levels, we investigated whether SP1 exerted its effect on LOXL2 through nuclear transfer. Nuclear and cytoplasmic separation experiments demonstrated that COL1A1 facilitated the translocation of SP1 from the cytoplasm to the nucleus in ovarian cancer cells (Fig. 5a). Immunofluorescence confirmed these results (Fig. 5b).

COL1A1 promotes the activation of the EGFR–MEK–ERK pathway by enhancing integrin signaling^17,18^, and the activation of the MEK–ERK signaling pathway can facilitate the nuclear translocation of transcription factors^19^. Thus, we hypothesized that COL1A1 activates EGFR–MEK–ERK signaling to promote SP1 translocation into the nucleus and stimulate LOXL2 transcription. Consequently, we first demonstrated that COL1A1 facilitates EGFR–MEK–ERK signaling pathway activation (Supplementary Fig. 9b). Subsequently, we discovered that the MEK inhibitor U0126 effectively antagonized the activation of the MEK–ERK pathway induced by COL1A1 (Fig. 5c). In addition, the nuclear translocation of SP1 induced by COL1A1 was effectively reversed by U0126 (Fig. 5d,e), but the total level of SP1 remained unchanged (Supplementary Fig. 9c). At the same time, the dramatically increased LOXL2 expression and transcriptional activity induced by COL1A1 was also reversed by U0126 (Fig. 5f,g).

To confirm the molecular mechanism by which COL1A1 promotes metastasis in vitro, we used a mouse xenograft model of ovarian cancer. We injected CAOV3 cells labeled with luciferase into the abdominal cavities of mice and randomly divided them into two groups: COL1A1 and IgG groups. The COL1A1 group received intraperitoneal injections of COL1A1 (2 μg per 200 μl) every other day, while the IgG group received intraperitoneal injections of IgG (2 μg per 200 μl) on the same schedule. After 2 weeks, we randomly divided the COL1A1 group into two subgroups. U0126 (15 mg/kg) or DMSO was intraperitoneally injected twice per week, and the mice were euthanized after 4 weeks. We found that the elevated peritoneal metastasis, tumor numbers and tumor weight induced by COL1A1 were reversed by U0126 (Fig. 5h–j and Supplementary Fig. 9d). The IHC assay showed that the increases in p-ERK and LOXL2, as well as the nuclear translocation of SP1 induced by COL1A1, were retrieved by U0126 (Fig. 5k and Supplementary Fig. 9e). In conclusion, our results indicated that COL1A1 regulates LOXL2 expression via EGFR–MEK–ERK-dependent SP1 translocation.

### EGFR is identified as a binding protein of LOXL2

**Fig. 6:**
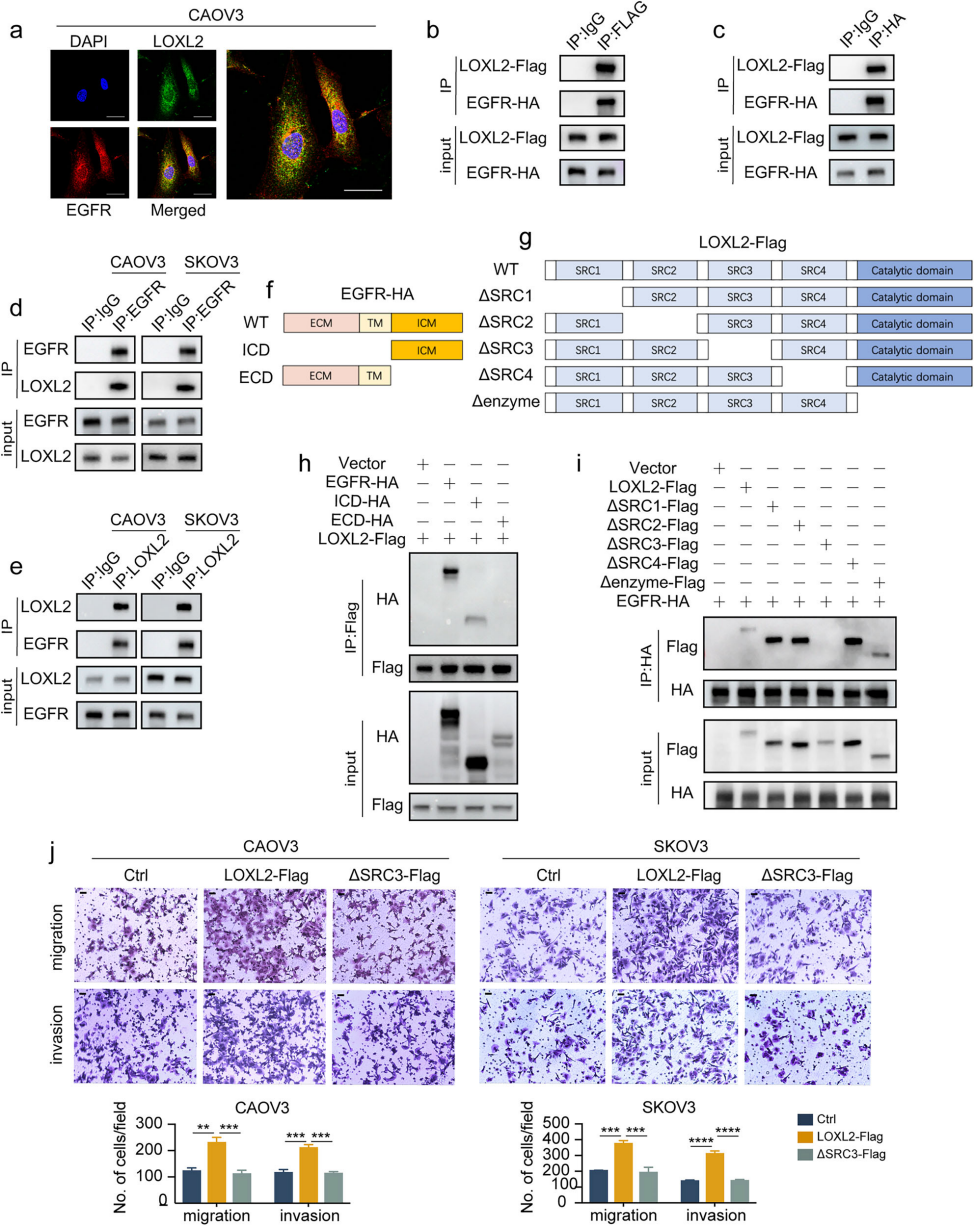
EGFR is identified as a binding protein of LOXL2. **a** Immunofluorescence assay for the colocalization of LOXL2 and EGFR. **b**,**c** The exogenous interaction between LOXL2 and EGFR was confirmed by an IP assay using a Flag (**b**) or HA (**c**) antibody. **d**,**e** The endogenous interaction between LOXL2 and EGFR was confirmed by an IP assay using an EGFR (**d**) or LOXL2 (**e**) antibody. **f**,**g** A schematic representation of truncated constructs of EGFR (**f**) and LOXL2 (**g**). **h** The interaction between LOXL2 and different truncated constructs of EGFR was confirmed by IP experiments. **i** The interaction between EGFR and different truncated constructs of LOXL2 was confirmed by IP experiments. **j**, The role of WT LOXL2 and its truncated variants in promoting ovarian cancer migration and invasion was verified by a transwell assay. Scale bar, 100 μm. Data are representative of at least three independent experiments. *P < 0.05, **P < 0.01, ***P < 0.001 and ****P < 0.0001.

To explore the potential mechanism of LOXL2 in ovarian cancer, we identified LOXL2-binding proteins through immunoprecipitation (IP)–MS. Interestingly, we found that EGFR, which is upstream of the MEK–ERK signaling pathway, binds LOXL2. The colocalization of LOXL2 and EGFR was confirmed by immunofluorescence (Fig. 6a). We exogenously transfected Flag-tagged LOXL2 and HA-tagged EGFR into 293T cells, and IP experiments confirmed exogenous binding between LOXL2 and EGFR (Fig. 6b,c). Endogenous interactions between LOXL2 and EGFR were also confirmed in CAOV3 and SKOV3 cells (Fig. 6d,e). To further explore the specific binding sites between LOXL2 and EGFR, we designed truncated EGFR and LOXL2, as shown in Fig. 6f,g. We found that the intracellular domains of EGFR and the SRC3 domain of LOXL2 were necessary for their interaction (Fig. 6h,i). In addition, compared to wild-type (WT) LOXL2, the ΔSRC3-LOXL2 truncated protein is unable to promote the metastasis of ovarian cancer (Fig. 6j and Supplementary Fig. 10a). Taken together, our results show that the interaction between LOXL2 and EGFR affects ovarian cancer metastasis.

### LOXL2 promotes EGFR–MEK–ERK signaling by protecting EGFR from lysosomal degradation

**Fig. 7:**
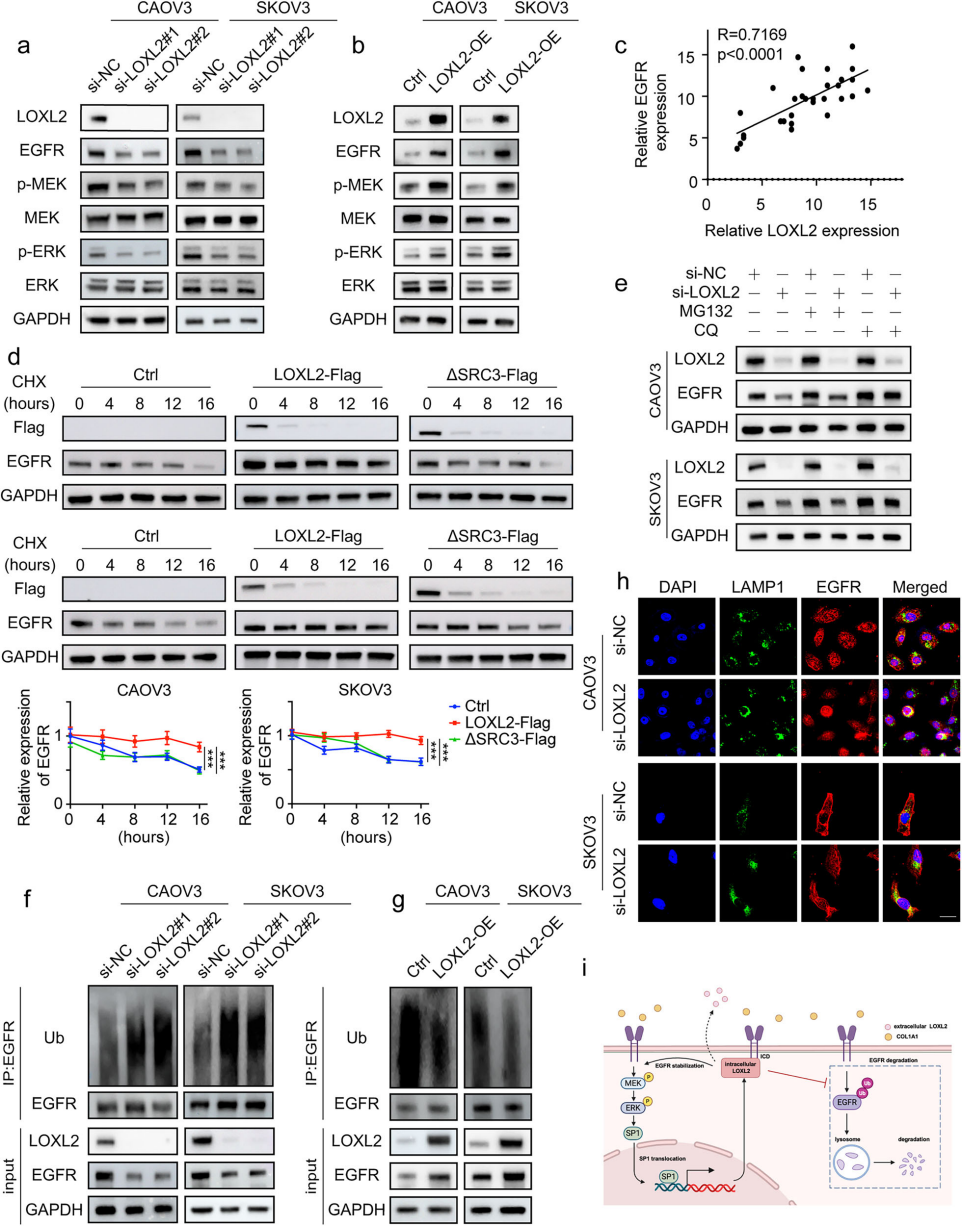
LOXL2 promotes EGFR–MEK–ERK signaling by protecting EGFR from lysosomal degradation. **a**,**b** EGFR, p-MEK, MEK, p-ERK and ERK expression was determined by immunoblot analysis in ovarian cancer cells with LOXL2 downregulation (**a**) or LOXL2 upregulation (**b**). **c** Immunohistochemical experiments for the relationship of LOXL2 and EGFR. **d** Analysis of LOXL2 and EGFR expression by immunoblotting in CAOV3 and SKOV3 cells transfected with LOXL2, ΔSRC3-LOXL2 or control plasmids, followed by CHX (50 μg/ml) treatment. **e** Ovarian cancer cells with or without LOXL2 downregulation were treated with 50 μM MG132 or 20 μM CQ for 10 h. LOXL2 and EGFR expression was detected by immunoblot analysis. **f**,**g** CAOV3 and SKOV3 cells with LOXL2 downregulation (**f**) or overexpression (**g**) were immunoprecipitated with an EGFR antibody followed by immunoblot analysis with a ubiquitin antibody. **h** CAOV3 and SKOV3 cells were transfected with LOXL2 siRNA or NC with the colocalization of LAMP1 and EGFR confirmed by immunofluorescence. **i** A mechanistic illustration showing the COL1A1–LOXL2 loop in ovarian cancer. Data are representative of at least three independent experiments. *P < 0.05, **P < 0.01, ***P < 0.001 and ****P < 0.0001.

By further investigating the relationship between LOXL2 and EGFR, we found that LOXL2 did not alter EGFR mRNA levels (Supplementary Fig. 11a,b), but did alter its protein expression (Fig. 7a,b). In addition, we examined the downstream signaling pathway of EGFR and found that LOXL2 promoted MEK–ERK signaling activation (Fig. 7a,b). IHC experiments further confirmed the positive correlation between LOXL2 and EGFR expression (Fig. 7c). Therefore, we postulate that LOXL2 regulates EGFR expression via post-translational modifications. To explore EGFR protein stability, we utilized cycloheximide (CHX) to inhibit intracellular protein synthesis. Our findings revealed that in both CAOV3 and SKOV3 cells, LOXL2 knockdown markedly decreased EGFR protein stability (Supplementary Fig. 11c), while LOXL2 overexpression significantly increased EGFR protein stability. However, overexpression of ΔSRC3-LOXL2 failed to enhance EGFR protein stability (Fig. 7d). Furthermore, we observed restored EGFR protein expression in cells with downregulated LOXL2 expression after application of the lysosomal autophagy inhibitor chloroquine (CQ), but not the proteasome inhibitor MG132 (Fig. 7e). In addition, ubiquitination experiments confirmed that EGFR ubiquitination was increased upon LOXL2 knockdown (Fig. 7f) and decreased when LOXL2 expression was elevated (Fig. 7g). To clarify how LOXL2 regulates EGFR degradation, we knocked down LOXL2 in ovarian cancer cells and analyzed EGFR localization using markers for early endosomes (Rab5), late endosomes (Rab7) and lysosomes (LAMP1). We found that LOXL2 knockdown increased the colocalization of EGFR with the lysosomal marker LAMP1 (Fig. 7h and Supplementary Fig. 11d,e). These results collectively suggested that LOXL2 enhanced the EGFR–MEK–ERK pathway by protecting EGFR from lysosomal degradation.

## Discussion

Our previous study revealed that COL1A1 derived from fibroblasts facilitated the metastasis of ovarian cancer cells^6^. In the current study, we observed a positive correlation between COL1A1 and LOXL2 expression in the ascites fluid of patients with ovarian cancer. High LOXL2 expression levels were associated with high-risk clinical parameters, such as FIGO stage, lymph node metastasis, CA125 levels and ascites volume, and were indicative of poorer survival prognosis. In addition, LOXL2 expression is higher in metastatic ovarian cancer tissues than in primary ovarian cancer tissues. Our in vitro and in vivo experiments confirmed that COL1A1 promotes ovarian cancer metastasis by enhancing LOXL2 expression. Importantly, LOXL2 silencing dramatically reversed the increased metastasis induced by COL1A1 treatment in a xenograft mouse, suggesting that LOXL2 is a downstream target of COL1A1. As an independent predictor of survival, LOXL2 may have the potential to be used clinically as a biomarker associated with patient prognosis and as a therapeutic target in metastatic ovarian cancer.

However, the molecular mechanism underlying how COL1A1 enhances LOXL2 protein levels remains unknown. Using RT–qPCR and dual-luciferase reporter assays, we discovered that COL1A1 increased the LOXL2 transcriptional activity. Analysis of the LOXL2 promoter region using JASPAR revealed multiple binding domains rich in SP1 transcription factor. SP1, one of the earliest-identified transcription factors^20^, plays a role in various cancers by regulating gene transcription in the nucleus^21,22^. In colon cancer, SP1 effectively activates DDX39B transcription, thereby enhancing cancer cell proliferation, migration and invasion^23^. Targeting and inhibiting SP1 in ovarian cancer cells can effectively suppress cancer cells^24^. Our findings indicate that COL1A1 facilitates the nuclear translocation of SP1, enabling it to bind to the −871 to −618 segment of the LOXL2 promoter region, thereby promoting LOXL2 transcription and expression.

COL1A1 encodes the pro-α1 chain of type I collagen and is often highly expressed in tumor tissues^25^. As a secreted protein, COL1A1 activates corresponding signaling pathways within cells, promoting cancer cell migration and invasion and increasing cellular resistance to chemotherapeutic drugs^26,27^. The EGFR–MEK–ERK pathway has been reported to induce SP1 phosphorylation, facilitating the transcription of downstream target genes^28^. In liver cancer cells, activation of the ERK signaling pathway promotes the phosphorylation of SP1, thereby inducing Rfng transcription^29^. In pancreatic cancer cells, inhibition of EGFR activity suppresses p-SP1 levels, consequently reducing Cox-2 expression^30^. Our research revealed that COL1A1 activates the intracellular EGFR–MEK–ERK pathway, facilitating the nuclear translocation of SP1 and enhancing LOXL2 transcription. Moreover, the elevation of LOXL2 and SP1 expression in the nucleus induced by COL1A1 treatment was retrieved by the MEK inhibitor U0126 in vitro and in vivo.

Multiple studies have demonstrated a significant oncogenic role of LOXL2 in cancer^31^. However, clinical trials have revealed that the anti-LOXL2 antibody, simtuzumab, has no notable impact on the survival of patients with pancreatic and colorectal cancer^32,33^. The failure of these clinical trials can be attributed to simtuzumab specifically targeting extracellular LOXL2. Thus, the intracellular mechanisms of LOXL2 are crucial for cancer progression^34^. LOXL2, located in the nucleus, interacts with Snail to suppress E-cadherin expression, thereby facilitating the epithelial–mesenchymal transition (EMT) in cancer progression^35,36^, inducing EMT by interacting with Snail1 and synergistically inhibiting E-cadherin expression^37^. LOXL2 inhibit proteasome-mediated protein degradation of integrin α5 and integrin β1 in renal cancer cells^38^. LOXL2 interacts with MARCKSL1 to inhibit MARCKSL1-induced apoptosis and to promote breast cancer cell growth^39^. Therefore, we explored the mechanisms by which intracellular LOXL2 promotes ovarian cancer metastasis to develop more effective therapeutic strategies for LOXL2-targeted treatment.

Through IP experiments, we screened for the downstream binding proteins of LOXL2 and identified epidermal growth factor receptor (EGFR). EGFR is a promising candidate for therapeutic targeting in ovarian cancer given its positivity in 70% of ovarian cancers and its correlation with an unfavorable prognosis^40,41^. Efforts to inhibit EGFR activity have been made using monoclonal antibodies and EGFR-tyrosine kinase inhibitors (TKIs)^42^. The monoclonal anti-EGFR-antibody cetuximab can effectively inhibit cell growth, suppress angiogenesis and impair the invasive capabilities of ovarian cancer cells^43,44^. Furthermore, in vitro experiments have confirmed that EGFR-TKIs inhibit the transmission of intracellular receptor signals, thereby suppressing the proliferation of ovarian cancer cells^45^. Nevertheless, several phase I–II studies on ovarian cancer failed to show significant therapeutic improvement with either cetuximab or EGFR-TKIs^46,47^. This may be because primary and long-term cultured ovarian cancer cells exhibit a high degree of resistance to EGFR-targeted therapies^45^. Therefore, exploring the combination therapy of monoclonal antibodies or EGFR-TKIs with chemotherapy or PARP inhibitors represents a direction for future research^48,49^. In this study, we found that LOXL2 affected the protein level of EGFR, but not the mRNA level. We confirmed that LOXL2 binds to the intracellular region of EGFR through the SRC3 domain, protecting EGFR from lysosomal degradation and promoting its stability, thereby activating the downstream MEK–ERK pathway and forming a positive feedback loop. Therefore, LOXL2 inhibitors may inhibit EGFR expression and enhance the inhibitory effects of monoclonal antibodies or EGFR-TKIs on cancer.

In summary, our experiments confirmed that COL1A1 promotes the nuclear translocation of SP1 by activating the EGFR–MEK–ERK pathway and enhancing LOXL2 transcription. Increased intracellular LOXL2 binds to EGFR, increasing its stability and further activating the MEK–ERK pathway. Secreted LOXL2, an extracellular copper-dependent amine oxidase, catalyzes the crosslinking of elastin and collagen, which is essential for the remodeling and stabilization of the extracellular matrix^50^ (Fig. 7i). Our findings first demonstrate a positive feedback loop between COL1A1 and LOXL2, providing a potential approach for blocking ovarian cancer metastasis. Nevertheless, the precise mechanism by which COL1A1 activates the EGFR–MEK–ERK pathway remains to be elucidated and represents a promising direction for future investigation.

## Supplementary information

Supplementary Information

## Data Availability

All data created and analyzed during this current work are included in this Article and its Supplementary Information.
