# Supplementary material for: COL1A1-induced LOXL2 promotes ovarian cancer metastasis via a feedback loop upon inhibiting EGFR lysosomal degradation
Source: Exp Mol Med. 2026 Mar 11;58(3):864–78. doi: 10.1038/s12276-026-01675-6 (PMC13049134; doi:10.1038/s12276-026-01675-6)
Supplement: Supplementary file 1 — Supplementary Information [file 12276_2026_1675_MOESM1_ESM.pdf]

COL1A1-induced LOXL2 promotes ovarian cancer metastasis via a feedback loop upon inhibiting EGFR lysosomal degradation

Zhangjin Shen[123#], Lingkai Gu[2#], Mengxia Zheng[2#], Yuwan Liu [2], Shanliang Shang[1], Yunshan Zhu[13*], Weiguo Lu[23*]

Supplementary Figures

Supplementary Figure1

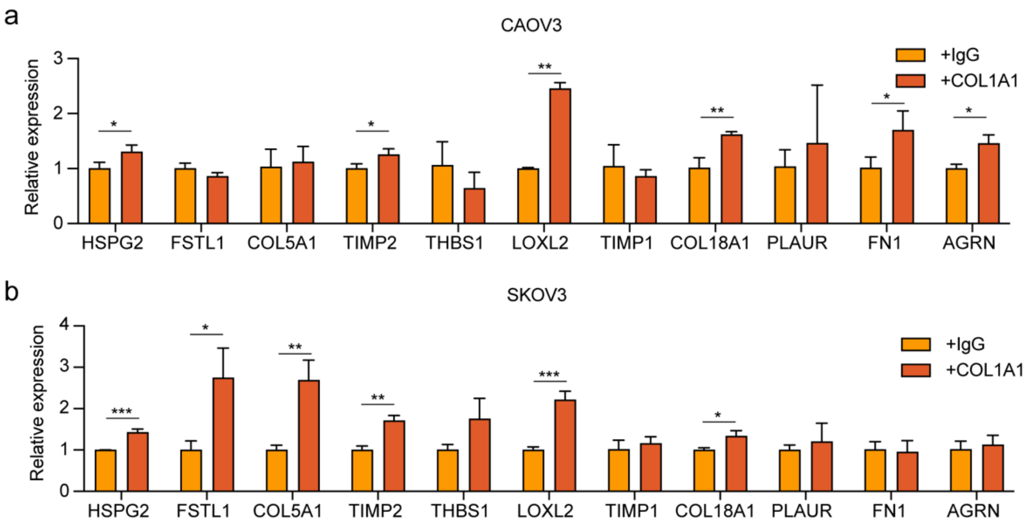

Supplementary Fig. 1 a. RT-qPCR analysis of differentially expressed proteins in CAOV3 cells incubated with COL1A1 or IgG. **b.** RT-qPCR analysis of differentially expressed proteins in SKOV3 cells incubated with COL1A1 or IgG.

Data are representative of at least three independent experiments. *p < 0.05, **p < 0.01, ***p < 0.001， ****p < 0.0001

Supplementary Figure2

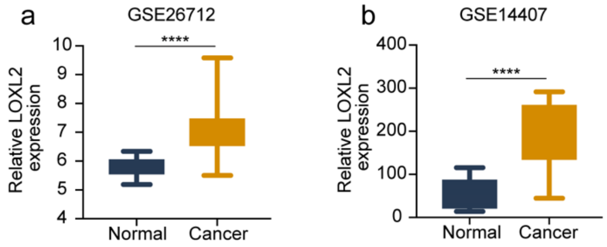

Supplementary Fig. 2 a-b. Relative expression of LOXL2 in ovarian cancer tissues and normal ovarian tissues from GEO datasets.

Supplementary Figure3

a

univariate analysis and multivariate analysis of prognostic factors for overall survival in ovarian cancer patients

variable	Univariate analysis		Multivariate analysis	
	**HR (95% CI)**	**P value**	**HR (95% CI)**	**P value**
Age(years) (≤50 vs. >50)	1.362(0.757-2.452)	0.303		
Serum CA125 (U/ml) (<500 vs. ≥500)	1.573(0.916-2.702)	0.101		
FIGO stage (I/II vs. III/IV)	3.619(1.541-8.496)	**0.003**	0.947(0.31-2.894)	0.923
Ascitic fluid volume (ml) (<500 vs. ≥500)	1.906(1.108-3.278)	**0.020**	1.301(0.746-2.268)	0.354
Lymph node metastasis (negative vs. positive)	5.793(1.803-18.608)	**0.003**	3.439(0.77-15.419)	0.107
Tumor diameter(cm) (<8 vs. ≥8)	0.903(0.526-1.55)	0.711		
Primary surgery (Optimal vs. Suboptimal)	3.828(1.949-7.516)	**<0.001**	3.251(1.626-6.502)	**0.001**
LOXL2 expression (low vs. high)	3.645(1.966-6.757)	**<0.001**	3.033(1.602-5.742)	**0.001**

HR: hazard ratio, 95%CI：95% confidence interval

b

univariate analysis and multivariate analysis of prognostic factors for progression free survival in ovarian cancer patients

variable	Univariate analysis		Multivariate analysis	
	**HR (95% CI)**	**P value**	**HR (95% CI)**	**P value**
Age(years) (≤50 vs. >50)	1.064(0.681-1.664)	0.785		
Serum CA125 (U/ml) (<500 vs. ≥500)	1.582(1.029-2.432)	**0.037**	1.022(0.644-1.621)	0.927
FIGO stage (I/II vs. III/IV)	3.035(1.64-5.616)	**<0.001**	1.693(0.670-4.274)	0.265
Ascitic fluid volume (ml) (<500 vs. ≥500)	1.596(1.032-2.467)	**0.035**	1.229(0.775-1.950)	0.381
Lymph node metastasis (negative vs. positive)	3.21(1.602-6.434)	**0.001**	1.524(0.536-4.332)	0.429
Tumor diameter(cm) (<8 vs. ≥8)	0.917(0.597-1.407)	0.691		
Primary surgery (Optimal vs. Suboptimal)	2.459(1.352-4.472)	**0.003**	1.917(1.035-3.550)	**0.038**
LOXL2 expression (low vs. high)	2.17(1.395-3.402)	**0.001**	1.796(1.127-2.863)	**0.014**

HR: hazard ratio, 95%CI：95% confidence interval

Supplementary Fig. 3 a. Univariate analysis and multivariate analysis of prognostic factors for overall survival in ovarian cancer patients. **b.** Univariate analysis and multivariate analysis of prognostic factors for progression free survival in ovarian cancer patients.

Supplementary Figure4

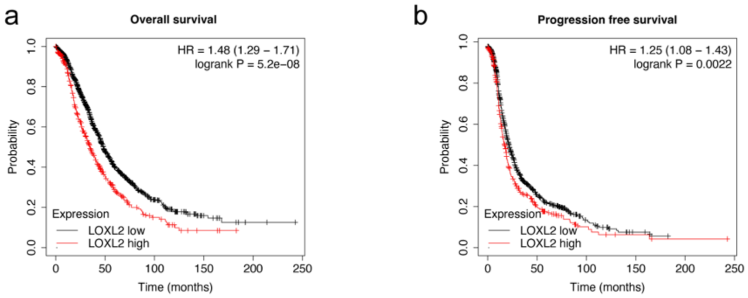

Supplementary Fig. 4 a. Kaplan-Meier analysis of overall survival of ovarian cancer patients from TCGA database. **b.** Kaplan-Meier analysis of progression free survival of ovarian cancer patients from TCGA database.

Supplementary Figure5

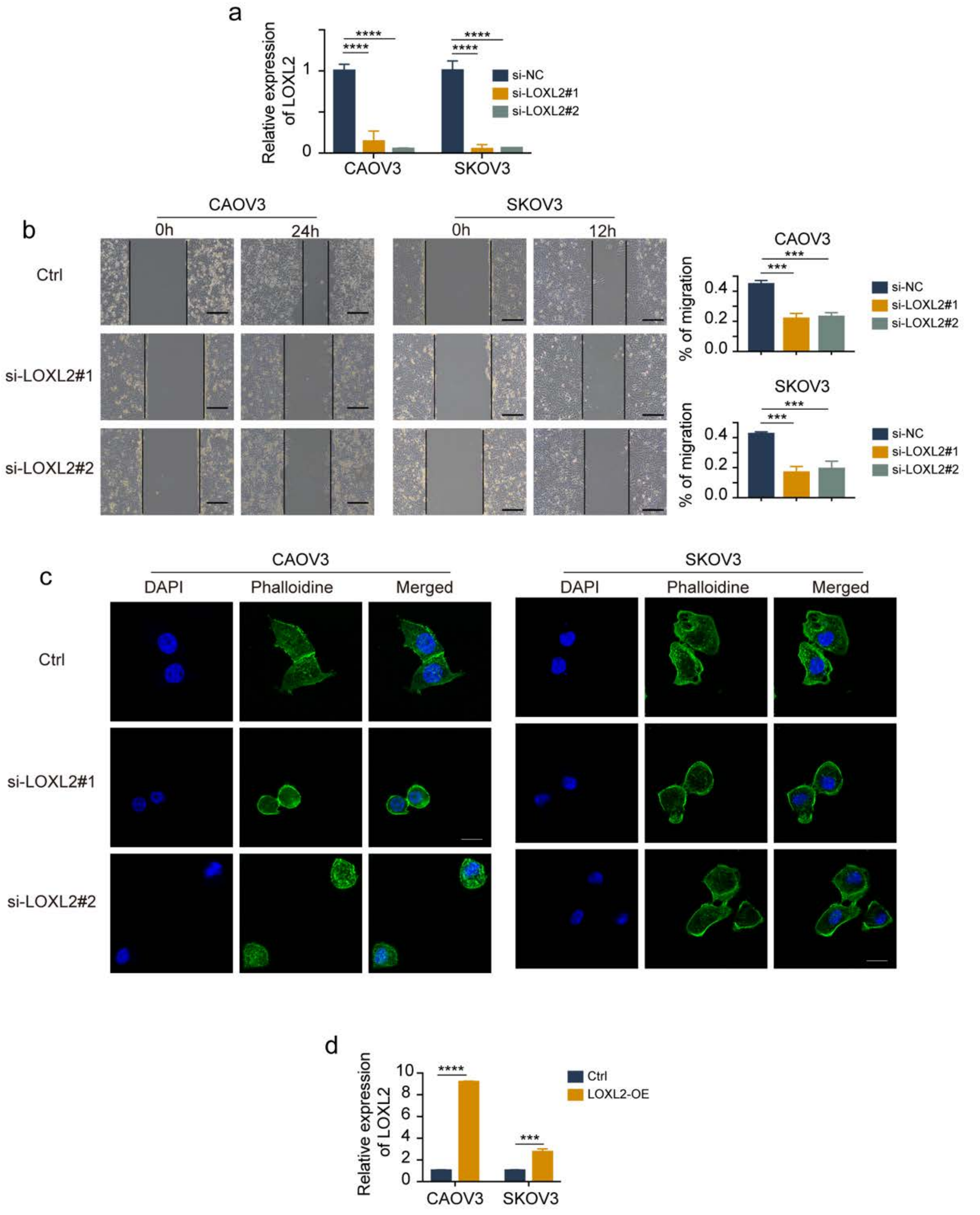

Supplementary Fig. 5 a. CAOV3 and SKOV3 cells were transfected with two LOXL2 siRNAs or negative control, LOXL2 expression was determined by RT-qPCR. **b**. CAOV3 and SKOV3 cells were transfected with two LOXL2 siRNAs or negative control. Cellular migration was detected by wound healing assay. Scale bar, 50 μm. **c.** Cellular structure of CAOV3 and SKOV3 cells transfected with LOXL2 siRNAs or negative control were detected by phalloidin staining. Scale bar, 50 μm. **d.**

CAOV3 and SKOV3 cells were transfected with LOXL2 plasmid or negative control, LOXL2 expression was determined by RT-qPCR.

Data are representative of at least three independent experiments. *p < 0.05, **p < 0.01, ***p < 0.001， ****p < 0.0001

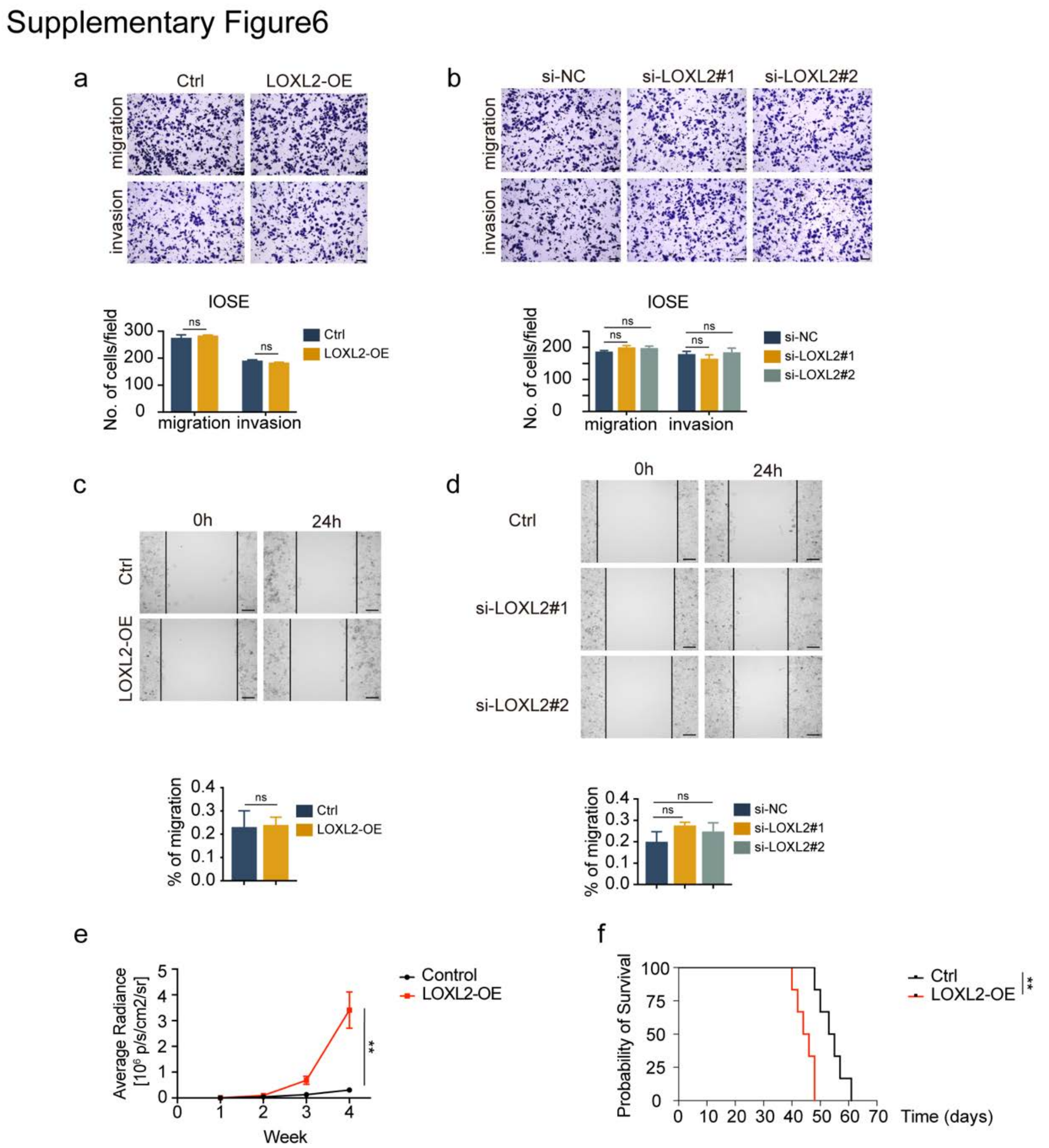

Supplementary Fig. 6 a. IOSE cells were transfected with LOXL2 overexpressing plasmid or empty plasmid; cellular migration and invasion were detected by transwell assay. Scale bar, 100 µm. **b.** IOSE cells were transfected with two LOXL2 siRNAs or

negative control, cellular migration and invasion were detected by transwell assay. Scale bar, 100 μm. **c.** IOSE cells were transfected with LOXL2 overexpressing plasmid or empty plasmid, cellular migration was detected by wound healing assay. Scale bar, 100 μm. **d.** IOSE cells were transfected with two LOXL2 siRNAs or negative control, cellular migration was detected by wound healing assay. Scale bar, 100 μm. **e.** Weekly luminescence quantification of peritoneal metastasis. **f.** Survival curve of nude mice that had been intraperitoneal injected with CAOV3-LOXL2-luc or CAOV3-control-luc cells.

Data are representative of at least three independent experiments. *p < 0.05, **p < 0.01, ***p < 0.001, ****p < 0.0001

Supplementary Figure7

a

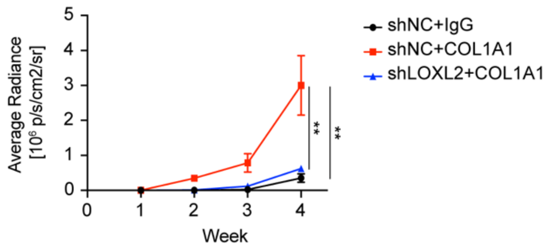

Supplementary Fig. 7 a. Weekly luminescence quantification of peritoneal metastasis. **p < 0.01.

Supplementary Figure8

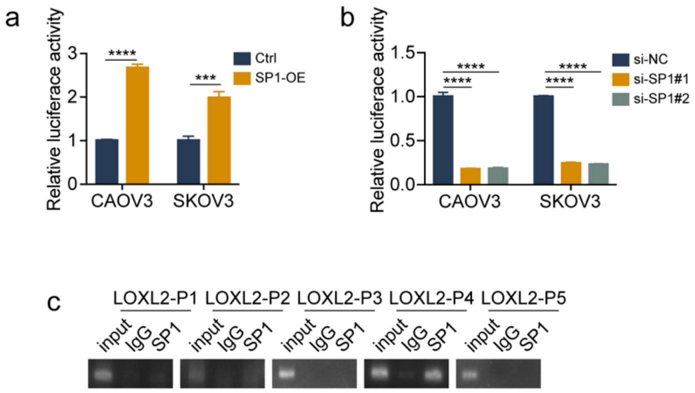

Supplementary Fig. 8 a. CAOV3 and SKOV3 cells were transfected with SP1 plasmid or negative control, respectively. LOXL2 transcription were determined by dual-luciferase reporter assays. **b.** CAOV3 and SKOV3 cells were transfected with SP1 siRNA or negative control, respectively. LOXL2 transcription were determined by dual-luciferase reporter assays. **c.** Enrichment of SP1 or IgG at the LOXL2 promoter was assessed by ChIP assay.

Data are representative of at least three independent experiments. *$p < 0.05$, **$p < 0.01$, ***$p < 0.001$， ****$p < 0.0001$

Supplementary Figure9

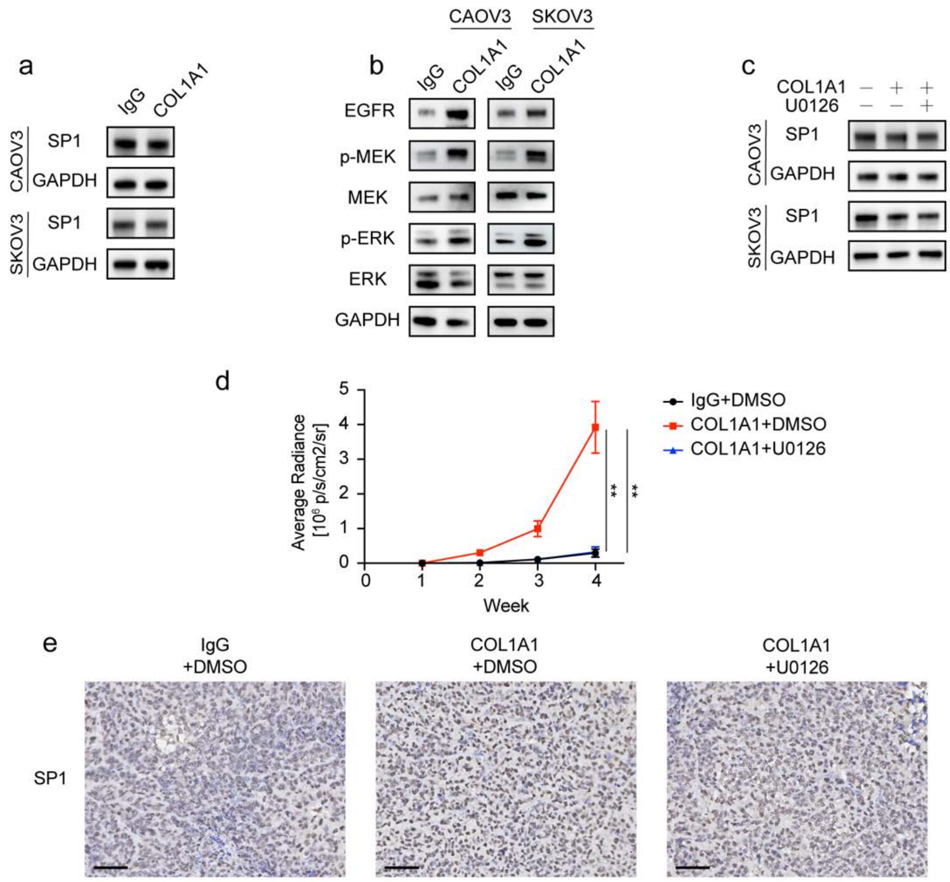

Supplementary Fig. 9 a. CAOV3 and SKOV3 cells were incubated with IgG or COL1A1, respectively. SP1 expression were determined by immunoblot analysis. **b.** CAOV3 and SKOV3 cells were incubated with IgG or COL1A1, respectively. EGFR, p-MEK, MEK, p-ERK and ERK expressions were determined by immunoblot analysis. **c.** CAOV3 and SKOV3 cells were treated with COL1A1, COL1A1 plus U0126, and negative control, respectively. SP1 expression were determined by immunoblot analysis. **d.** Weekly luminescence quantification of peritoneal metastasis. **e.** Representative IHC staining images of SP1 in xenograft mouse model.

**p < 0.01.

Supplementary Figure10

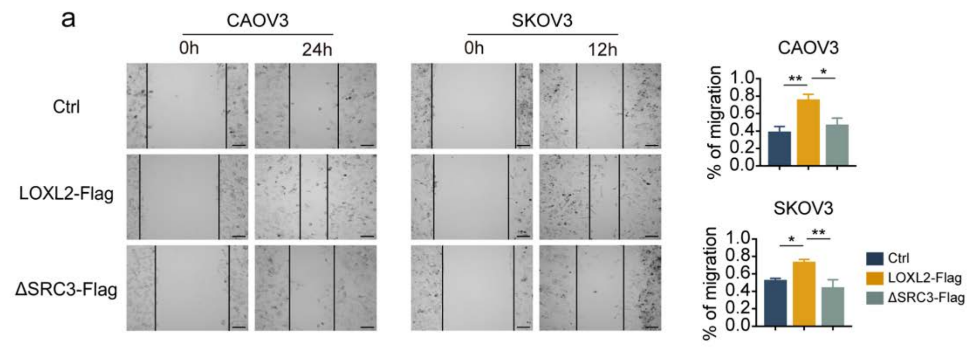

Supplementary Fig. 10 a. The role of wild-type LOXL2 and its truncated variants in promoting ovarian cancer migration was verified by wound healing assay. Scale bar, 100 μm.

Data are representative of at least three independent experiments. *p < 0.05, **p < 0.01, ***p < 0.001， ****p < 0.0001

Supplementary Figure11

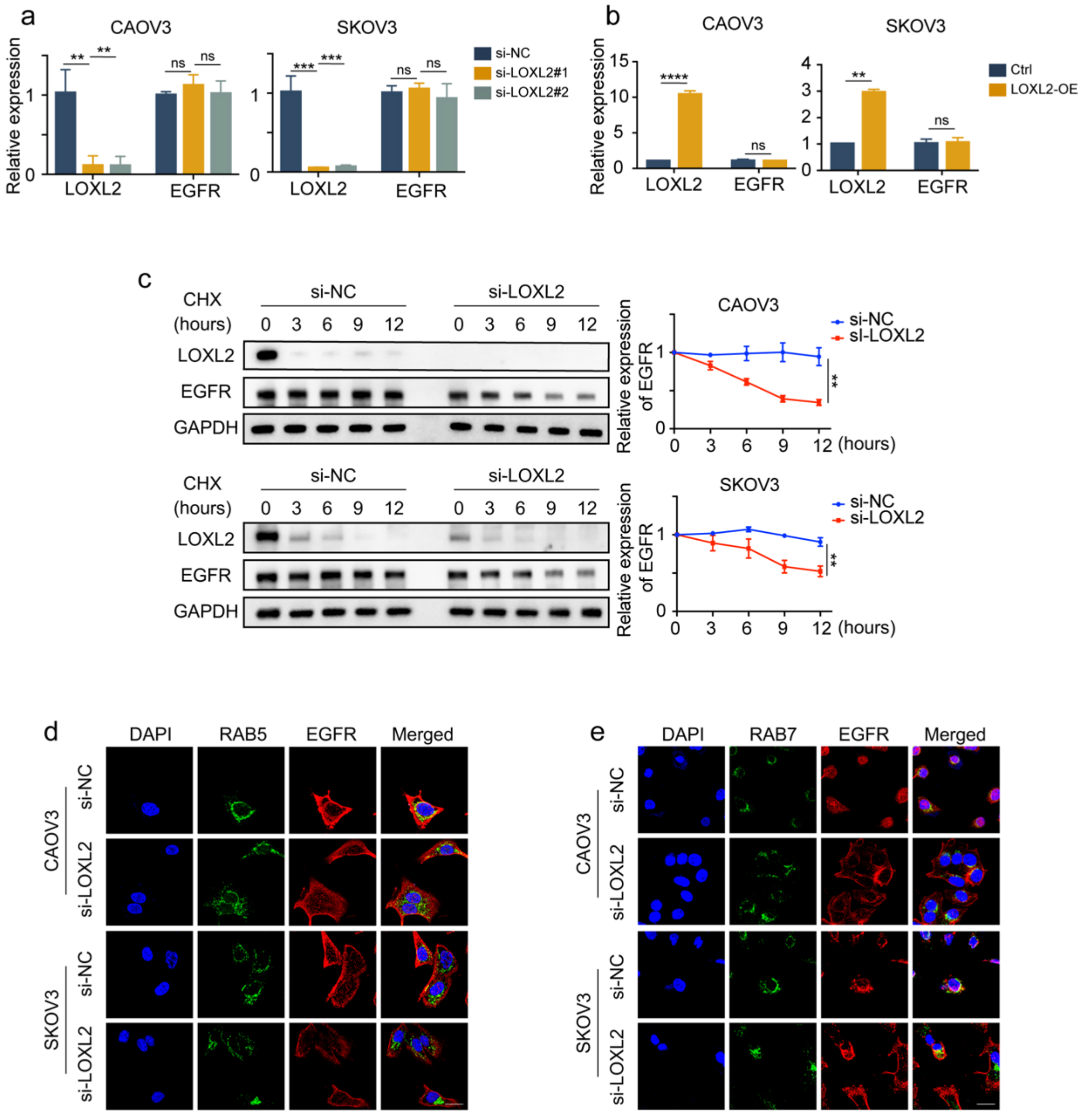

Supplementary Fig. 11 a. CAOV3 and SKOV3 cells were transfected with LOXL2 siRNAs or negative control, LOXL2 and EGFR expression was determined by RT-qPCR. **b.** CAOV3 and SKOV3 cells were transfected with LOXL2 plasmids or negative control, LOXL2 and EGFR expression was determined by RT-qPCR. **c.** CAOV3 and SKOV3 cells with LOXL2 downregulation were treated with 50 μg/ml of cycloheximide (CHX) for different hours. LOXL2 and EGFR expressions were detected by immunoblot analysis. **d.** CAOV3 and SKOV3 cells were transfected with LOXL2 siRNA or negative control, colocalization of RAB5 and EGFR by

immunofluorescence. **e.** CAOV3 and SKOV3 cells were transfected with LOXL2 siRNA or negative control, colocalization of RAB7 and EGFR by immunofluorescence. Data are representative of at least three independent experiments. $*p < 0.05$, $**p < 0.01$, $***p < 0.001$， $****p < 0.0001$

Supplementary Tables

Supplementary Table 1 Clinicopathologic characteristics of ovarian cancer associated with LOXL2 protein expression

Case No.	Age (years)	Serum CA125(U/mL)	Figo stage	Ascitic fluid volume(mL)	Lymph node metastasis	Tumor diameter (cm)	Primary surgery
1	63	281.9	III	1500	+	8.4	Suboptimal
2	56	2722	III	100	+	10.5	Suboptimal
3	50	66.6	II	0	-	8	Optimal
4	66	49.9	II	500	+	10.9	Optimal
5	60	9	II	50	-	7.2	Optimal
6	41	61.1	I	20	-	5.9	Optimal
7	64	32.2	II	50	-	3.5	Optimal
8	45	45.1	I	0	-	7.1	Optimal
9	48	320	I	50	-	4	Optimal
10	47	2295	I	500	-	11.1	Optimal
11	46	28.1	II	200	+	8.8	Optimal
12	50	18.3	I	0	-	10	Optimal
13	64	264.6	III	300	+	3.3	Optimal
14	65	409.4	III	0	+	5.6	Optimal
15	45	155.6	II	100	-	16.8	Optimal
16	57	79.2	I	0	-	5.9	Optimal
17	50	63.1	I	100	-	9	Optimal
18	66	170.6	III	100	+	18.3	Optimal

19	63	429.6	IV	150	+	5.1	Optimal
20	43	132.7	III	500	+	14.9	Optimal
21	34	28.1	I	50	-	8.7	Optimal
22	52	483.1	III	200	+	4.5	Optimal
23	24	236.2	III	1000	+	10.9	Optimal
24	42	4870	III	500	+	5.5	Suboptimal
25	36	112.6	I	50	-	6.8	Optimal
26	49	74.9	III	100	-	7	Optimal
27	49	221.5	III	50	+	13.6	Optimal
28	47	202.6	III	500	+	6.8	Optimal
29	44	89.5	III	50	+	8.5	Optimal
30	50	196.9	III	200	+	6.2	Optimal
31	61	342.6	III	100	+	4.3	Optimal
32	62	539.6	III	200	+	16.4	Optimal
33	57	130.1	II	0	-	6.7	Optimal
34	64	622.3	III	200	+	5.6	Suboptimal
35	55	364.8	III	1000	+	15.7	Optimal
36	54	1054	II	0	+	10.8	Optimal
37	69	44.4	II	100	-	6.5	Optimal
38	65	122.6	III	50	+	16.7	Optimal
39	43	880.2	III	100	+	7	Optimal
40	60	76.9	III	200	+	13.3	Optimal
41	58	2006	III	300	+	13	Optimal
42	52	566.8	III	100	+	8.1	Optimal
43	57	643	III	100	+	4	Optimal

44	53	1497	III	500	+	6	Optimal
45	47	1652	III	5000	+	14.3	Optimal
46	63	2365	II	1000	+	13.4	Optimal
47	54	863.4	III	100	+	3.9	Suboptimal
48	42	388	III	1200	+	8.8	Optimal
49	60	788.7	III	300	+	10.8	Optimal
50	51	546.1	III	100	+	7.3	Optimal
51	66	70.1	II	0	-	10.5	Optimal
52	45	335.8	III	200	+	7.8	Optimal
53	62	727.6	III	3000	+	5.8	Optimal
54	72	36.5	II	0	-	8.1	Optimal
55	44	648.5	III	150	+	11.2	Optimal
56	52	297	II	50	-	5	Optimal
57	43	3488	III	200	+	3.3	Optimal
58	47	120.9	III	50	+	5.5	Optimal
59	41	1154	III	100	+	8.9	Optimal
60	50	812.3	III	50	+	10.3	Optimal
61	53	35.3	II	100	+	9.3	Optimal
62	52	725.8	III	500	+	13.5	Suboptimal
63	49	956.6	III	50	+	9.8	Optimal
64	63	48.6	III	500	+	5.6	Optimal
65	45	137.5	III	50	-	5.6	Optimal
66	45	986.8	III	50	+	7.1	Optimal
67	64	492.1	III	100	+	3	Optimal
68	40	214.9	III	200	+	7.5	Optimal

69	39	82.9	III	50	+	5	Optimal
70	67	639.3	III	200	+	4.5	Optimal
71	65	363.6	III	1800	+	3.6	Optimal
72	68	269	III	100	+	8.4	Optimal
73	43	832.6	III	500	+	5.3	Optimal
74	40	73.9	III	200	+	6.5	Optimal
75	39	3159	III	3500	+	4.9	Optimal
76	67	373.9	I	100	-	5.5	Optimal
77	54	1260	III	400	+	9.9	Optimal
78	61	74.5	II	800	+	10.3	Optimal
79	46	392.7	III	600	+	9.3	Optimal
80	46	714.5	III	100	+	5.5	Optimal
81	51	581	II	2000	-	16	Optimal
82	64	402.9	III	300	+	10	Optimal
83	59	595.6	III	1000	+	10	Optimal
84	51	980.7	III	1800	+	14	Optimal
85	62	318.7	II	50	-	7	Optimal
86	47	1822	III	500	+	5.8	Suboptimal
87	57	366	III	500	+	6	Optimal
88	50	1136	III	400	+	11.5	Optimal
89	48	11696	III	800	+	11.2	Optimal
90	69	153.3	III	50	+	7.9	Optimal
91	43	3940	III	2000	+	12.3	Optimal
92	68	12.2	I	0	-	2.5	Optimal
93	55	4595	III	3000	+	14.8	Suboptimal

94	35	1676	III	6500	+	5.9	Suboptimal
95	49	480.3	III	1500	+	5	Optimal
96	49	475.6	III	100	+	11.6	Optimal
97	51	204.3	III	100	+	8.2	Optimal
98	63	1745	III	300	+	2.4	Optimal
99	40	645.7	III	300	+	13.2	Optimal
100	51	1406	III	200	+	11.3	Suboptimal
101	62	2155	III	500	+	15.4	Optimal
102	52	2469	III	100	+	10.2	Optimal
103	55	717.4	III	100	+	13.6	Suboptimal
104	54	1763	III	1000	+	5.3	Optimal
105	61	4374	III	500	+	8.7	Optimal
106	47	2217	III	5000	+	12.7	Optimal
107	61	1097	III	3000	+	4.9	Optimal
108	41	241.1	II	200	+	5	Optimal
109	54	103.2	III	3000	+	11.3	Suboptimal
110	53	1245	IV	6000	+	0.6	Suboptimal
111	61	1714	III	300	+	12.5	Optimal
112	46	719	II	200	-	7.5	Optimal
113	51	932.3	III	600	+	9.8	Optimal
114	50	466.8	III	4000	+	8.2	Optimal
115	68	363.1	III	100	+	12.8	Optimal
116	77	103.9	III	100	+	4.8	Optimal
117	52	898.6	Ⅳ	100	+	5.5	Optimal
118	66	108.5	III	0	+	3.7	Optimal

119	66	4388	III	5000	+	5.5	Optimal
120	65	3118	IV	7000	+	13.7	Optimal
121	44	4014	III	800	+	6.7	Optimal
122	65	186.2	III	150	+	6.2	Optimal
123	52	286.6	III	200	+	10.7	Optimal
124	47	450.3	III	7000	+	9.4	Suboptimal
125	66	153.9	IV	300	+	10.8	Suboptimal
126	63	247.3	III	100	+	5.8	Optimal
127	69	745.9	IV	1000	+	8.9	Optimal
128	46	1818	III	500	+	8.6	Optimal
129	53	18805	III	4500	+	5.5	Optimal

Supplementary Table 2 Sequences of siRNAs against specific targets in this study.

Item	Sequence	
LOXL2 shRNA	Target sequence	AGACATCCAGAAGAATTAC
LOXL2 siRNA-1	Sense (5’-3’)	GAGAGGACAUACAAUACCATT
	Antisense (5’-3’)	UGGUAUUGUAUGUCCUCUCTT
LOXL2 siRNA-2	Sense (5’-3’)	CCAGAAGAAUUACGAGUGUTT
	Antisense (5’-3’)	ACACUCGUAAUUCUUCUGGTT
SP1 siRNA-1	Sense (5’-3’)	GCAGACCUUUACAACUCAATT
	Antisense (5’-3’)	UUGAGUUGUAAAGGUCUGCTT
SP1 siRNA-2	Sense (5’-3’)	GCCGUUGGCUAUAGCAAAUTT
	Antisense (5’-3’)	AUUUGCUAUAGCCAACGGCTT
Ctrl siRNA	Sense (5’-3’)	UUCUCCGAACGUGUCACGUTT

	Antisense (5'-3')	ACGUGACACGUUCGGAGAATT

Supplementary Table 3 Sequences of primers in this study.

Item	Sequence	
LOXL2	Forward (5'-3')	AGGACATTCGGATTCGAGCC
	Reverse (5'-3')	CTTCCTCCGTGAGGCAAAC
GAPDH	Forward (5'-3')	GGAGCGAGATCCCTCCAAAAT
	Reverse (5'-3')	GGCTGTTGTCATACTTCTCATGG
HSPG2	Forward (5'-3')	CCAAATGCGCTGGACACATTC
	Reverse (5'-3')	CGGACACCTCTCGGAACTCT
FSTL1	Forward (5'-3')	GAGCAATGCAAACCTCACAAG
	Reverse (5'-3')	CAGTGTCCATCGTAATCAACCTG
COL5A1	Forward (5'-3')	GCCCGGATGTCGCTTACAG
	Reverse (5'-3')	AAATGCAGACGCAGGGTACAG
TIMP2	Forward (5'-3')	GCTGCGAGTGCAAGATCAC
	Reverse (5'-3')	TGGTGCCCGTTGATGTTCTTC
THBS1	Forward (5'-3')	AGACTCCGCATCGCAAAGG
	Reverse (5'-3')	TCACCACGTTGTTGTCAAGGG
TIMP1	Forward (5'-3')	CTTCTGCAATTCCGACCTCGT
	Reverse (5'-3')	ACGCTGGTATAAGGTGGTCTG
COL18A1	Forward (5'-3')	CAGTGGACACACTTAGCCCTC
	Reverse (5'-3')	GCGGCATTCTCTGGAACTCC
PLAUR	Forward (5'-3')	TGTAAGACCAACGGGGATTGC
	Reverse (5'-3')	AGCCAGTCCGATAGCTCAGG

FN1	Forward (5'-3')	CGGTGGCTGTCAGTCAAAG
	Reverse (5'-3')	AAACCTCGGCTTCCTCCATAA
AGRN	Forward (5'-3')	GTCCTGCGTCTGCAAGAAGAG
	Reverse (5'-3')	CTCGCATTCGTTGCTGTAGG
SP1	Forward (5'-3')	TGGCAGCAGTACCAATGGC
	Reverse (5'-3')	CCAGGTAGTCCTGTCAGAACTT
EGFR	Forward (5'-3')	AGGCACGAGTAACAAGCTCAC
	Reverse (5'-3')	ATGAGGACATAACCAGCCACC
LOXL2-P1	Forward (5'-3')	TGAGATGTGGTTGGCAGTTGGG
	Reverse (5'-3')	AGCTACTCGGGAAGCTGAGGC
LOXL2-P2	Forward (5'-3')	GTGGTTTTCTGTATCTCAAGACACT
	Reverse (5'-3')	CCACCTTGACTCCTGTGAGC
LOXL2-P3	Forward (5'-3')	GGGAATGTCCAGGTGAAAGTCT
	Reverse (5'-3')	GAGGCAGCGAGCTGCAAAAC
LOXL2-P4	Forward (5'-3')	CGCCTTTCAGGTGACAGTCTACTTC
	Reverse (5'-3')	CTGGAATGGTAGGGCTGGGAT
LOXL2-P5	Forward (5'-3')	GCGAGATGGGTGCAAGTTTCTGA
	Reverse (5'-3')	GCCTCCGGCCTCTTTTGTTGTAAT

Supplementary Table 4 Antibodies used in this study.

Antigens	Manufacturer	Application
LOXL2 (A4708)	Abclonal, China	1:1000 for WB
SP1 (21962-1-AP)	Proteintech, USA	1:2000 for WB,<br>1:200 for IHC

		1:100 for IF<br>5 µg per reaction for ChIP
p-ERK (#4370)	CST, USA	1:2000 for WB<br>1:500 for IHC
ERK (#4695)	CST, USA	1:1000 for WB
GAPDH (db106)	Diagbio, China	1:2000 for WB
LaminB1 (db35)	Diagbio, China	1:1000 for WB
LOXL2 (ab96233)	Abcam, USA	1:500 for IHC<br>1:200 for IF in cell<br>1:500 for IF in tissue
LOXL2 (ab314140)	Abcam, USA	5 µg per reaction for IP
HA (#3724)	CST, USA	1:1000 for WB
α-SMA(#19245)	CST, USA	1:600 for IF in tissue
Flag (#14793)	CST, USA	1:1000 for WB
p-MEK1/2 (#9154)	CST, USA	1:1000 for WB
MEK1/2 (#4694)	CST, USA	1:1000 for WB
Ubiquitin (AF1705)	Beyotime, China	1:1000 for WB
EGFR（#4267）	CST, USA	1:1000 for WB<br>1:100 for IF<br>1:50 for IHC<br>5 µg per reaction for IP
RAB5（11947-1-AP）	Proteintech, China	1:200 for IF
RAB7（84741-1-RR）	Proteintech, China	1:500 for IF
LAMP1（84658-5-RR）	Proteintech, China	1:200 for IF
